# Partially hydrolyzed, whey-based infant formula with six human milk oligosaccharides, *B. infantis* LMG11588, and *B. lactis* CNCM I-3446 is safe, well tolerated, and improves gut health: a staged analysis of a randomized trial

**DOI:** 10.3389/fnut.2025.1628847

**Published:** 2025-07-23

**Authors:** Jean-Charles Picaud, Olivier Claris, Mercedes Gil-Campos, Ignacio Salamanca De La Cueva, Luc Cornette, Philippe Alliet, André Léké, Mireille Castanet, Hugues Piloquet, Virginie de Halleux, Delphine Mitanchez, Yvan Vandenplas, Pierre Maton, Frank Jochum, Dirk Olbertz, Sergio Negre Policarpo, Luca Lavalle, Cecilia Fumero, Paula Rodriguez-Garcia, Janne Marie Moll, Irma Silva-Zolezzi, Boutaina Zemrani, Nicholas P. Hays, Norbert Sprenger, Javier Miranda-Mallea

**Affiliations:** ^1^Department of Neonatology, Hôpital de La Croix-Rousse, Lyon, France; ^2^CarMen Laboratory, INSERM, INRA, Université Claude Bernard Lyon 1, Pierre-Bénite, Lyon, France; ^3^Department of Neonatology, Hôpital Femme Mère Enfants, Bron, France; ^4^Paediatric Metabolism Unit, Reina Sofia University Hospital, University of Córdoba, IMIBIC, CIBEROBN, Córdoba, Spain; ^5^Department of Pediatrics, Instituto Hispalense de Pediatria, Sevilla, Spain; ^6^Department of Neonatology, AZ Sint-Jan Hospital, Brugge, Belgium; ^7^Department of Pediatrics, Jessa Hospital, Hasselt, Belgium; ^8^Neonatal Medicine and Intensive Care, Centre Hospitalier Universitaire Amiens Picardie, Amiens, France; ^9^CIC INSERM U1404, Department of Pediatrics, Rouen University Hospital Charles Nicole, Rouen, France; ^10^Child Chronic Disease Service, Centre Hospitalier Universitaire de Nantes, Nantes, France; ^11^Neonatology Division, Centre Hospitalier Universitaire de Liège – Centre Hospitalier Universitaire de la Citadelle, Liège, Belgium; ^12^Service de Néonatologie, Centre Hospitalier Universitaire de Tours, Tours, France; ^13^KidZ Health Castle, Vrije Universiteit Brussel, UZ Brussel, Brussels, Belgium; ^14^Service Néonatal, Clinique CHC-Montlégia, Liège, Belgium; ^15^Department of Pediatrics, Evangelisches Waldkrankenhaus Spandau, Berlin, Germany; ^16^Department of Neonatology, Klinikum Südstadt Rostock, Rostock, Germany; ^17^Department of Pediatrics, Hospital Quironsalud, Valencia, Spain; ^18^Clinical Research Unit, Nestlé Research, Lausanne, Switzerland; ^19^Cmbio, Copenhagen, Denmark; ^20^Clinical and Nutritional Research Unit, Nestlé Product Technology Center – Nutrition, Vevey, Switzerland; ^21^Nestlé Institute of Health Sciences, Nestlé Research, Lausanne, Switzerland; ^22^Department of Pediatrics, Hospital Vithas, Valencia, Spain

**Keywords:** bifidobacteria, gastrointestinal tolerance, growth, gut health, microbiota

## Abstract

**Background and aims:**

Gut health and microbiome development are closely linked in early life, with human milk oligosaccharides (HMOs) playing a key role. This study reports results through 4 months of age from a trial evaluating an infant formula containing a synbiotic blend of HMOs and probiotics, focusing on growth, gastrointestinal (GI) tolerance, and gut health biomarkers from birth to 15 months.

**Materials and methods:**

Healthy infants aged ≤14 days were randomized to receive either the experimental formula (SYN; control formula supplemented with six HMOs and two probiotics [*B. infantis*, *B. lactis*]) or the control formula (CTRL; partially hydrolyzed 100% whey-based formula). A non-randomized breastfed (BF) group served as a reference. The primary endpoint was weight gain velocity in SYN vs. CTRL through 4 months of age. Secondary endpoints included fecal outcomes (abundance of bifidobacteria, immune and gut health markers), GI tolerance, and adverse events (AEs).

**Results:**

The full analysis set (FAS) included 313 infants (118 in SYN, 114 in CTRL, and 81 BF), while the per-protocol population (PP) included 227 infants (84 in SYN, 84 in CTRL, and 59 BF). Weight gain velocity through 4 months in the SYN group was non-inferior to that in the CTRL group in both FAS and PP analyses (both *p* < 0.0001). Parent-reported GI tolerance and stool patterns were similar between SYN and CTRL groups through 4 months. At 3 months, *Bifidobacteria* abundance was significantly higher in the SYN group compared to the CTRL group (*p* = 0.004). Fecal pH was lower in the SYN group than in the CTRL group (*p* = 0.018) and more closely resembled that of the BF group. Immune and gut health markers were similar between the SYN and BF groups. No significant differences in AEs were observed across groups.

**Conclusion:**

The synbiotic-supplemented infant formula supported healthy, age-appropriate growth, good GI tolerance, and increased the abundance of beneficial *bifidobacteria* through 4 months of age.

**Clinical trial registration:**

https://clinicaltrials.gov/study/NCT04962594.

## 1 Introduction

Structurally diverse compounds, including essential nutrients, hormones, bacteria, immune components, and oligosaccharides, are present in human milk and serve various functions that impact infant growth and development (1). In human milk, oligosaccharide concentration ranges between 5 and 15 g/L, while cow’s milk, which is the primary raw material for making infant formula, contains very low levels at approximately 0.05 g/L (2). Human milk also contains bacteria; it is estimated that breastfed infants consume approximately 10e4–10e6 bacteria per day, with the majority of the species belonging to *Staphylococcus*, *Streptococcus*, *Lactobacillus*, and *Bifidobacterium* (1). Oligosaccharides and bacteria present in human milk influence nutrient availability for gut bacteria, enhance colonization efficiency, alter host–microbe interactions, and promote the growth of beneficial bacteria, thereby impacting the long-term health of the infant (1). Given the importance of oligosaccharides and beneficial bacteria for infant health and their absence in cow’s milk used in infant formulas, researching the inclusion of manufactured oligosaccharides, which are structurally identical to human milk oligosaccharides (HMOs), and probiotics in infant formula is essential to provide these benefits when breastfeeding is not possible.

Recently conducted preclinical and clinical studies offer valuable insights into the health impacts of HMO- and synbiotic-supplemented formulas (3–7). Several clinical trials reported an increase in the relative abundance of *Bifidobacteria* among infants receiving formula supplemented with HMOs compared to infants receiving control formula (6) and noted, for example, shared features of mucus enrichment and tyrosine degradation between the 5-HMO supplemented group and the breastfed (BF) infants (8), as well as higher secretory IgA upon HMO formula feeding compared to controls (9). In both clinical settings and preclinical fermentation models using infant stool, a lack of HMO-metabolizing bifidobacteria is observed in many infants, resulting in an altered gut ecology characterized by higher pH and lower age-appropriate concentrations of short-chain fatty acids (SCFAs) (10–12). In an *ex vivo* colonic infant stool fermentation model, an increase in SCFA production was observed in tested fecal samples from all infants when a blend of six HMOs, equivalent to the HMOs tested here, was combined with an HMO-metabolizing probiotic, *B. longum* subsp. *infantis* LMG 11588, compared to the resulting SCFA production from individual ingredients, suggesting that combining HMOs with specific HMO-metabolizing probiotics may benefit all infants (11). Additional presence of the widely used probiotic *B. lactis* (CNCM I-3446) did not affect the observed SCFA production (11). As there are remaining questions about safety and suitability (often required by regulatory authorities) as well as gut and microbiome benefits related to synbiotic-supplemented infant formula, clinical studies are needed to better understand synbiotic blends used to supplement infant formulas.

The primary objective of this study was to evaluate the effect of a unique blend of six HMOs and two probiotic strains, with a composition adapted to the infant’s age during the first 15 months of life, on weight gain velocity from enrollment to 4 months of age in healthy infants. Our key secondary objective was to assess fecal bifidobacteria abundance at 3 months of age. Our other secondary objective were to assess gastrointestinal (GI) tolerance and markers of gut and immune health. Here, we report the first staged endpoint analysis covering data collected up to 4 months of infant age.

## 2 Participants and methods

### 2.1 Study design and population

This double-blind, randomized, controlled trial, with a non-randomized BF reference group, was conducted at 18 centers in Belgium, France, Germany, and Spain from November 2021 to October 2024. The study included several planned, staged end-point analyses and concluded when the last infant completed the 15-month clinic visit. Infants were enrolled into the study if they were aged between 0 and 14 days and met the following criteria: healthy, full-term birth (≥37 weeks of gestation); birth weight ≥2,500 g and ≤4,500 g; BF infants must have been exclusively breastfed since birth, and their parents must have decided to continue with exclusive breastfeeding at least through 4 months of age; and formula-fed infants must have been exclusively consuming and tolerating a cow’s milk infant formula, and their parents must have independently decided against breastfeeding before study enrollment. Infants were excluded from the study for the following reasons: they had conditions requiring infant feedings other than those specified in the protocol; there was evidence of major congenital malformations, systemic or congenital infections (e.g., syphilis), or previous or ongoing severe laboratory or medical abnormalities; they had received or were presently receiving medications or probiotic supplements; or they had ongoing or past participation in another interventional trial.

### 2.2 Randomization procedure and blinding

Formula-fed infants who met the eligibility criteria were randomized to the SYN or CTRL groups using a dynamic allocation algorithm with a 1:1 allocation ratio stratified by center, sex (male/female), and delivery mode (vaginal/cesarean section). Randomization was performed using Medidata Randomization Trial Supply Management (New York, NY, United States). BF infants were not randomized. Given that this was a double-blind trial with individual coding, the identity of the specific formula was concealed from everyone involved in the study, including participants, study staff, contract research organization staff, and the sponsor.

### 2.3 Intervention and study formulas

A graphical overview of the study design is shown in Supplementary Figure S1. The total duration of the study intervention was 15 months. The study formulas were both made with partially hydrolyzed whey-based protein (in order to promote digestive comfort) and staged according to the age of the infant (1st age infant formula [IF]: 0 to <6 months; 2nd age follow-up formula [FUF]: 6 to <12 months; 3rd age growing up milk [GUM]: 12 to 15 months). The SYN and CTRL formulas were identical, except that the SYN formula was supplemented with a blend of six HMOs and *B. longum* subsp. *infantis* (*B. infantis*) LMG11588 plus *B. animalis* subsp. *lactis* (*B. lactis*) CNCM I-3446 (also known as BL818), while CTRL was not. *B. lactis* CNCM I-3446 was included in the SYN group to further enrich it with a well-known probiotic. The six manufactured HMOs in the SYN group were 3-fucosyllactose (3-FL), 2′-fucosyllactose (2’FL), 2′-3-difucosyllactose (DFL), lacto-N-tetraose (LNT), 3′sialyllactose (3’SL), and 6′sialyllactose (6’SL), and the amount and ratio between the HMOs changed from IF to FUF to GUM formulas as the infants aged. In a previous trial using formulas containing a blend of 5 HMOs, we observed similar effects with 2.5 g/L and 1.5 g/L HMOs in reconstituted formula (9). In this study, we used 1.5 g/L as the effective dose and added one additional HMO (3-FL). The final concentration of each of the individually added HMOs is well within the range of those in human milk. The composition of the study formula is summarized in Table 1. Products were dispensed in cartons of six cans, each with a unique code. Study formulas were fed orally, ad libitum, and intake varied according to the infant’s age, weight, and appetite. The study formulas were similar in appearance and taste.

**Table 1:** Composition of study formulas used in the experimental and control study^a^.

Formula	Control Formula (CTRL)	Experimental Formula (SYN)
1^st^ age starter infant formula: fed from enrollment (≤14 days) up to age 6 months		
Description	Partially hydrolyzed 100% whey-based infant formula	Partially hydrolyzed 100% whey-based infant formula supplemented with 6 HMOs and two probiotics
Composition	67 kcal/100 mL reconstituted formula 1.3 g whey protein 7.55 g carbohydrates 3.5 g fat	Identical composition as control
Probiotic content	None	1 × 10^6^ CFU/g *B. lactis* 5 × 10^5^ CFU/g *B. infantis*
HMO content	None (1.77 g/L of lactose was added to compensate for the addition of HMOs in the experimental formula)	1.77 g/L of an HMO blend consisting of 2’FL (49% of total HMO content), DFL (7%), 3-FL (14%), LNT (16%), 3’SL (6%), and 6’SL (8%)
2^nd^ age follow-up formula (FUF): fed from age 6 months up to 12 months		
Description	Partially hydrolyzed 100% whey-based FUF	Partially hydrolyzed 100% whey-based FUF supplemented with 6 HMOs and two probiotics
Composition	67 kcal/100 mL reconstituted formula 1.3 g whey protein 8.4 g carbohydrates 3.1 g fat	Identical composition as control
Probiotic content	None	1 × 10^6^ CFU/g *B. lactis* 5 × 10^5^ CFU/g *B. infantis*
HMO content	None (0.87 g/L of lactose was added to compensate for the addition of HMOs in the experimental FUF)	0.87 g/L of an HMO blend consisting of 2’FL (30% of total HMO content), DFL (4%), 3-FL (30%), LNT (17%), 3’SL (12%), and 6’SL (5%)
3^rd^ age growing-up milk (GUM): fed from age 12 months up to 15 months		
Description	Partially hydrolyzed 100% whey-based GUM	Partially hydrolyzed 100% whey-based GUM supplemented with 6 HMOs and two probiotics
Composition	67 kcal/100 mL reconstituted formula 1.3 g whey protein 8.1 g carbohydrates 3.2 g fat	Identical composition as control
Probiotic content	None	1 × 10^6^ CFU/g *B. lactis* 5 × 10^5^ CFU/g *B. infantis*
HMO content	None (0.75 g/L of lactose was added to compensate for the addition of HMOs in the experimental GUM)	0.75 g/L of an HMO blend consisting of 2’FL (28% of total HMO content), DFL (4%), 3-FL (38%), LNT (10%), 3’SL (14%), and 6’SL (5%)

a 2’FL, 2′-fucosyllactose; 3-FL, 3-fucosyllactose; 3’SL, 3′-sialyllactose; 6’SL, 6′-sialyllactose; CFU, colony-forming unit; DFL, difucosyllactose; HMO, human milk oligosaccharide; LNT, lacto-N-tetraose.

### 2.4 Baseline

At baseline, study staff obtained demographic information and medical history through questionnaires. A comprehensive physical examination was also conducted with documentation of any relevant abnormalities (i.e., heart murmur, hypertension, abnormal heart rate, hearing loss, or marginal neurological defects) in the electronic case report form (eCRF).

### 2.5 Growth

While anthropometrics were collected at all visits, the primary endpoint of this trial was the velocity of weight gain, measured as the mean daily weight gain in grams per day from baseline to 4 months. Infants were weighed without clothing or a diaper on a calibrated electronic scale to the nearest 10 g. The weight measure was repeated until it was reproduced within 10 g, and the two weights were recorded and averaged. Weight gain in g/day was calculated as (weight at 4 months in g minus weight at baseline in g)/(age at 4 months in days minus age at baseline in days). Secondary endpoints included length (cm), head circumference (HC; cm), and corresponding sex- and age-specific z-scores. The length was measured to the nearest 0.1 cm using a standardized measuring board. If two consecutive measurements were not within 0.5 cm, the infant was measured a third time, and the two measures that were most closely aligned were documented and averaged. Similarly, HC was measured to the nearest 0.1 cm with a standard, non-elastic, plastic-coated measuring tape. If two consecutive measurements were not within 0.2 cm, the infant was measured a third time, and the two measures that were most closely aligned were documented and averaged. Using the World Health Organization (WHO) Child Growth Standards (13) as a reference, corresponding z-scores, including weight-for-age, length-for-age, weight-for-length, height-for-age, and BMI-for-age, were calculated.

### 2.6 GI tolerance and stool patterns

Stool patterns, including frequency, consistency, and difficulty passing stool, as well as GI symptoms and behaviors, were collected at each visit using a one-day retrospective GI Symptom and Behavior Record at baseline and a 3-day prospective GI Symptom and Behavior Diary completed at home for 3 days prior to each subsequent visit. For each bowel movement, the parent indicated whether the infant had difficulty passing stool. The mean number of reported stools was used to determine stool frequency. To determine stool consistency, a validated 4-point stool scale (0 = watery, 1 = loose, 2 = formed, 3 = hard) developed for infants was provided to the parents (14). Stool consistency was reported as the mean stool consistency of each reported stool and as the percentage of stools in each of the four scale categories. Frequency and amount of spitting-up/vomiting and flatulence, in addition to duration of crying and fussiness (<10 min, 10–30 min, >30 min-1 h, >1–2 h, >2–3 h, >3 h) and sleeping (0–8 h, 8–12, 12–16, 16–20, 20–24), were also collected. GI symptom burden was assessed using the Infant Gastrointestinal Symptom Questionnaire (IGSQ) (15), which was completed at each visit. The questionnaire is a standardized and validated instrument consisting of 13 questions assessing five domains (stooling, vomiting/spitting-up, crying, fussiness, and flatulence). Domain scores were summed to calculate a composite index score ranging from 13 to 65, with lower scores indicating lower GI symptom burden. Scores of 13–23, 24–30, and >30 represent good GI tolerance, some level of GI distress, and clinically meaningful GI distress, respectively (15).

### 2.7 Adverse events and medication use

Reported adverse events (AEs) and serious AEs (SAEs) were documented by each investigator as part of the eCRF, including the type, incidence, severity, seriousness, and relation to feeding. Data were also continuously collected from parents/legal representatives using an electronic Infant Illness Diary (IID) to capture the number of occurrences and the length of time an infant experienced the following symptoms: fever, respiratory tract infections, GI symptoms, and ear symptoms. For each parent-reported diagnosis, study physicians contacted the parent(s) or legal representative and determined whether the infant should be brought to the site for further evaluation (unscheduled visits). Once IID entries had been validated and confirmed by study physicians, this information was entered into the eCRF as an adverse event. Medication type and duration of use were recorded using the concomitant medication reporting form as part of the eCRF.

### 2.8 Fecal sample collection

Fecal samples were collected at home by the parents using a feces tube with an integrated spatula and without any buffers or additives, frozen in the home freezer (−20°C), and brought to the study site, keeping the samples frozen in an icepack during transport. Samples were collected up to 1 day after the baseline visit and within 3 days prior to study visits at 3, 6, 12, and 15 months. Samples were kept frozen at the site at −80°C until transferred every 3–4 months to the central lab for analysis.

### 2.9 Fecal bifidobacteria abundance

The relative abundance of bifidobacteria was determined using shotgun metagenomics, as previously described (9), with minor modifications. Briefly, deoxyribonucleic acid (DNA) was extracted from the fecal samples using the NucleoSpin Stool kit (Machery-Nagel) with bead beating at 2700 rpm for 5 min. DNA was normalized to 5 nM, followed by library preparation (Celero EZ DNA-seq Core Module Kit and Celero 96-Plex Adaptor Plate) using a DreamPrep NGS (Tecan) PCR amplification, double-sided magnetic bead size selection (AMPure XP, Beckman Coulter), and sequencing on a NovaSeq system (Illumina) with 2 × 150 bp read lengths. After adapter and host DNA removal, fecal microbiota diversity and composition were determined using CHAMP (16).

### 2.10 Probiotic *B. longum* subsp. i*nfantis* LMG11588 tracking

Strain-level resolution of *B. longum* subsp*. infantis* strains was performed as described in Capeding et al. (17). Briefly, single-nucleotide variants (SNVs) were profiled in each position of the *B. longum* subsp. infantis-specific genes (signature genes) in the samples with at least 250 reads mapping to the signature genes and where at least 10 of the signature genes were detected. Polymorphic signature gene SNVs underwent multiple sequence alignment and were used to build a phylogenetic tree. As phylogenetic references, the *B. longum* subsp. *infantis* LMG 11588 genome, 16 genomic sequences from the public database National Institutes of Health National Center for Biotechnology Information annotated as *B. longum* subsp. *infantis*, and one genome annotated as *B. longum* subsp. *longum* were included in the tree.

### 2.11 Fecal pH and organic acids

Fecal pH was assessed using pH indicator paper (pH range 1–10; Merck, Darmstadt, Germany) and fecal organic acids, including lactic acid, propionic acid, butyric acid, acetic acid, and valeric acid, were assessed using validated liquid chromatography–tandem mass spectrometry according to a modified published method (18). Quantitative changes from baseline and differences between feeding groups were determined. Data are reported per dry stool weight (to normalize results).

### 2.12 Fecal markers of immune and gut health

Fecal markers of immune response and gut barrier function were assessed by enzyme-linked immunosorbent assays (ELISA), including total secretory immunoglobulin (sIgA; Immundiagnostik AG, Bensheim, Germany), lipocalin-2 (BioVendor), calprotectin (Immunodiagnostik AG), and alpha-1-antitrypsin (AAT; BioVendor). Data are reported per dry stool weight (to normalize results).

### 2.13 Other outcomes

Bone quality measures, using speed of sound, and absenteeism were collected at different infant ages. Descriptive findings will be reported in a follow-up publication that describes the trial’s findings to 15 months. Approximately 1 mL of blood was voluntarily collected by trained staff during the 4-month site visit. Peripheral mononuclear cells were isolated from blood samples for immune cell profiling. These immune cell results will be reported separately.

### 2.14 Sample size

The sample size was calculated for the primary endpoint (weight gain velocity through 4 months of age) and the key secondary endpoint (*Bifidobacterium* abundance at 3 months of age) using data from prior studies. A non-inferiority boundary of −3 g/day was used to demonstrate non-inferiority in weight gain between the SYN and CTRL groups with a power of 90% and an *α*-level of 5%. It was estimated that 88 infants per arm would be needed for the primary endpoint. To demonstrate superiority in *Bifidobacterium* abundance in the SYN vs. CTRL group with an increase of 8% in relative abundance, a power of 90%, and an α-level of 5%, the estimated sample size was 94 infants. The power for individual hypotheses was chosen to be 90% to achieve an overall study power of 80% (0.9*0.9 = 0.81). Since the sample size for the key secondary endpoint was the highest, it was used to drive the total sample size calculation (=2*94/0.8). It was estimated that 236 infants would need to be randomized, anticipating an attrition rate of 20%.

### 2.15 Statistical analysis

The intention-to-treat (ITT) population comprised all randomized infants, whereas the full analysis (FAS) population included all infants except those who never received any of the assigned study product, failed to meet study entry eligibility criteria, or had no post-randomization data. The per-protocol (PP) population included all infants in the FAS without any departures from the protocol believed to impact the analyses of interest, which included non-compliance with formula (defined as <80% of study days from enrollment to age 4 months on assigned formula or breastmilk), use of concomitant foods prior to age 4 months, visits outside of the study visit window, and major deviations from study procedures (e.g., failure to obtain weight at baseline). The safety analysis set (SAS) consisted of all infants in the ITT population with documented use of at least one feeding of the study formula (or breast milk for the BF group), classified according to the feeding received irrespective of the randomization assignment. The primary endpoint of weight gain velocity was analyzed in both the FAS and PP populations. Clinical secondary endpoints were analyzed in the FAS population. Fecal microbiota profiles were also analyzed in the PP population, with sensitivity analyses conducted in a “sub-PP” population, consisting of all subjects in PP except those who had protocol deviations that may impact microbiome or gut tolerance-related outcomes, including consumption of prebiotic- and/or probiotic-containing food/supplements, and those in the SYN and CTRL groups who consumed breast milk. Safety analyses were conducted in the SAS population.

Descriptive statistics were calculated for all continuous and categorical variables. The primary endpoint of weight gain velocity was analyzed using analysis of covariance (ANCOVA), which corrected for baseline weight, sex, delivery mode, and study center. Non-inferiority was concluded if the lower bound of the two-sided 95% confidence interval (CI) for the model-based difference between the SYN and CTRL groups was above the non-inferiority margin of -3 g/day. Due to the non-randomized nature of the BF arm, continuous secondary endpoints were analyzed using the inverse probability of treatment weighting method, where the probability or propensity to breastfeed was derived from factors known to influence the choice of breastfeeding and included mother’s age, delivery method, highest level of education of the parents, number of people in the household, smoking status during pregnancy, current smoking status, country, and study center. All propensity score-weighted ANCOVA models were adjusted for study center and baseline value, as well as IGSQ, SCFA, and stool models, which were additionally adjusted for age. Anthropometric outcomes at each visit were further adjusted for sex and delivery mode. Pairwise comparisons between SYN, CTRL, and BF were adjusted for multiple comparisons using the Benjamini-Hochberg correction. Microbiome differences between the SYN and CTRL groups at 3 months were compared using models that corrected for site and baseline age, employing a linear regression framework with log-transformed relative abundances and a compositional bias correction based on LinDA (19). Where the demographic comparisons did not reveal differences in sex and delivery mode, these two parameters were excluded. Fisher’s exact test was used to test for differential prevalence of potentially pathogenic bacteria. Statistical tests were two-sided, using a significance level of 5%. All analyses were performed using SAS 9.4 (or higher) or R 4.1 (or higher).

### 2.16 Ethics details

The ethics committees at each institution in Belgium and Germany approved the trial. In France and Spain, the ethical approval was granted by a centralized committee. The study was conducted in compliance with the Declaration of Helsinki and the International Conference on Harmonization Guidelines for Good Clinical Practice. This trial was registered on ClinicalTrials.gov (NCT04962594), and the Consolidated Standards of Reporting Trials (CONSORT) checklist was followed for trial conduct and reporting (Supplementary Table S1). Parent(s)/legally authorized representative provided written informed consent prior to study enrollment.

## 3 Results

### 3.1 Study infants

**Figure 1:**
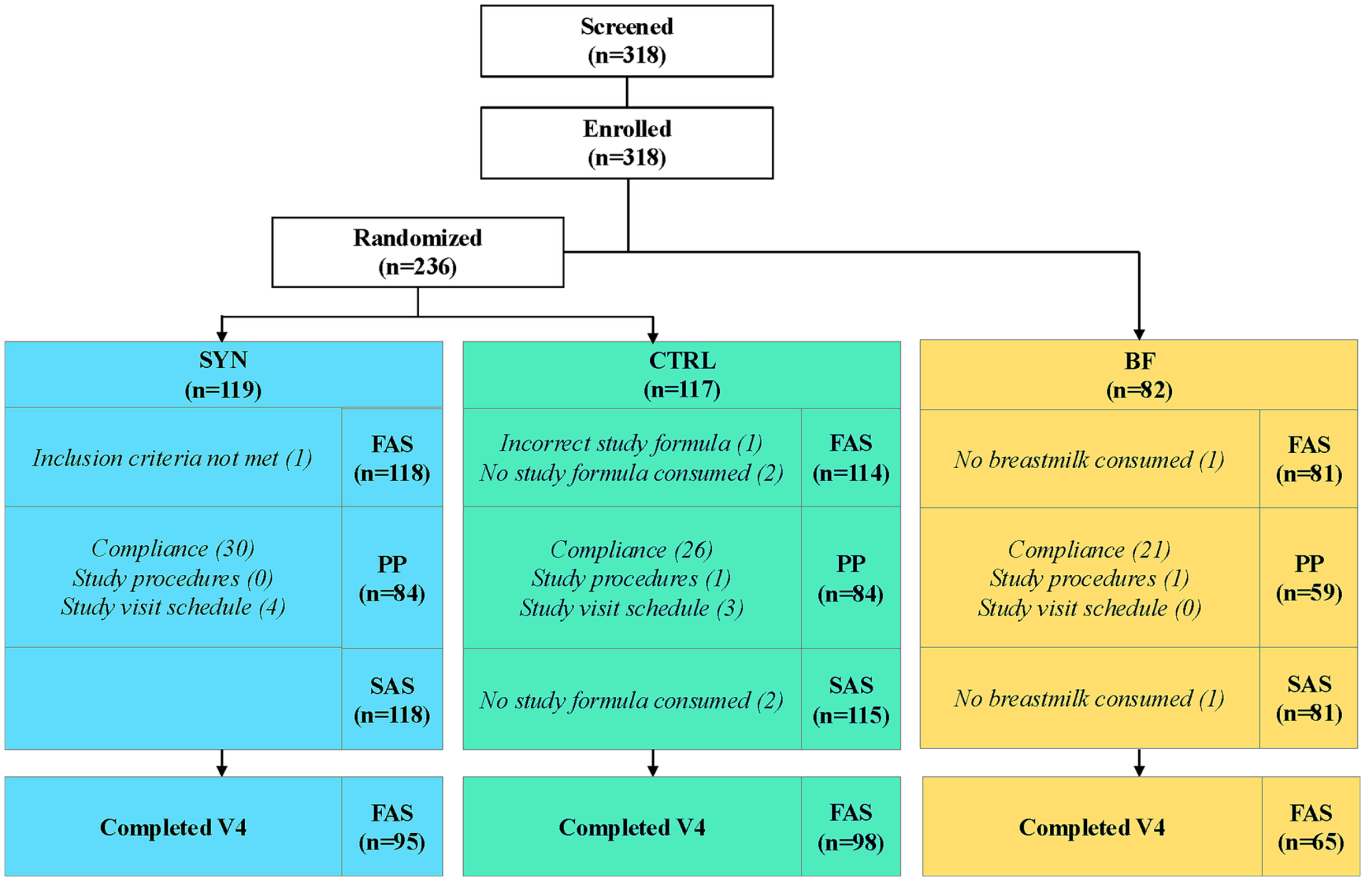
Infant disposition flowchart. Breastfed infants were enrolled in the breastfed reference group (BF), while formula-fed infants were randomized to receive either experimental formula (SYN) or control formula (CTRL). FAS, full analysis set; PP, per-protocol population; SAS, safety analysis set.

All screened infants (*n* = 318) were enrolled in the study (119 SYN, 117 CTRL, and 82 BF). The FAS included a total of 313 infants (118 SYN, 114 CTRL, and 81 BF) (Figure 1). Five infants were excluded from the FAS because they never consumed any of the assigned product (study formula or breast milk), received incorrect study formula, or failed to satisfy entry eligibility criteria. A total of 227 infants (84 SYN, 84 CTRL, and 59 BF) comprised the PP population after excluding subjects in FAS with major protocol deviations. The SAS consisted of 314 infants (118 SYN, 115 CTRL, and 81 BF) and included one infant who was excluded from the FAS due to receiving the incorrect study formula. A total of 258 infants completed the study through age 4 months: 95 SYN (80%), 98 CTRL (84%), and 65 BF (79%).

### 3.2 Baseline demographic and household characteristics

Infant baseline demographic and household characteristics, shown in Table 2, were similar between the SYN and CTRL groups, while differences in baseline age and maternal age were observed in the BF group compared to the SYN and CTRL groups.

**Table 2:** Baseline demographic and household characteristics for the full analysis set^a^.

Characteristic	SYN *n* = 118	CTRL *n* = 114	BF *n* = 81
Infant demographics			
Age at baseline, days^b^	6.9 ± 4.2	6.7 ± 4.6	8.6 ± 4.1
Gestational age, weeks	39.2 ± 1.2	39.1 ± 1.2	39.3 ± 1.2
Weight at baseline, kg	3.4 ± 0.4	3.4 ± 0.4	3.4 ± 0.4
Length at baseline, cm	50.8 ± 1.9	50.6 ± 2.0	51.2 ± 1.8
Head circumference at baseline, cm	35.0 ± 1.3	35.0 ± 1.2	35.2 ± 1.1
Infant sex, % female	49.2%	48.2%	44.4%
Type of delivery, % Cesarean	25.4%	25.4%	13.6%
APGAR at 5 min ≥ 9, %	94.1%	96.5%	97.5%
Household characteristics			
Daycare attendance, % no	83.9%	83.3%	90.1%
Number of siblings in household at birth^c^	1.2 ± 0.5	1.6 ± 0.8	1.4 ± 0.8
Maternal age, years^d^	32.4 ± 5.2	32.5 ± 5.2	34.1 ± 3.9
Maternal education, % completed associate degree or higher^d^	62.7%	53.5%	78.9%
Mother smokes, % no	88.1%	87.7%	96.3%

a Values are means ± SD unless otherwise specified. BF, breastfed group; CTRL, control formula-fed group; SYN, experimental formula-fed group.

b BF infants were significantly older than SYN and CTRL (*p* = 0.011 for both).

c SYN infants had fewer siblings compared to CTRL infants (*p* = 0.026).

d Mothers of BF infants were significantly older than mothers of SYN and CTRL infants (*p* = 0.020 for both) and were more educated than those of CTRL infants (*p* < 0.001). All *p*-values were adjusted for multiple comparisons using the Benjamini-Hochberg correction.

### 3.3 Growth

**Figure 2:**
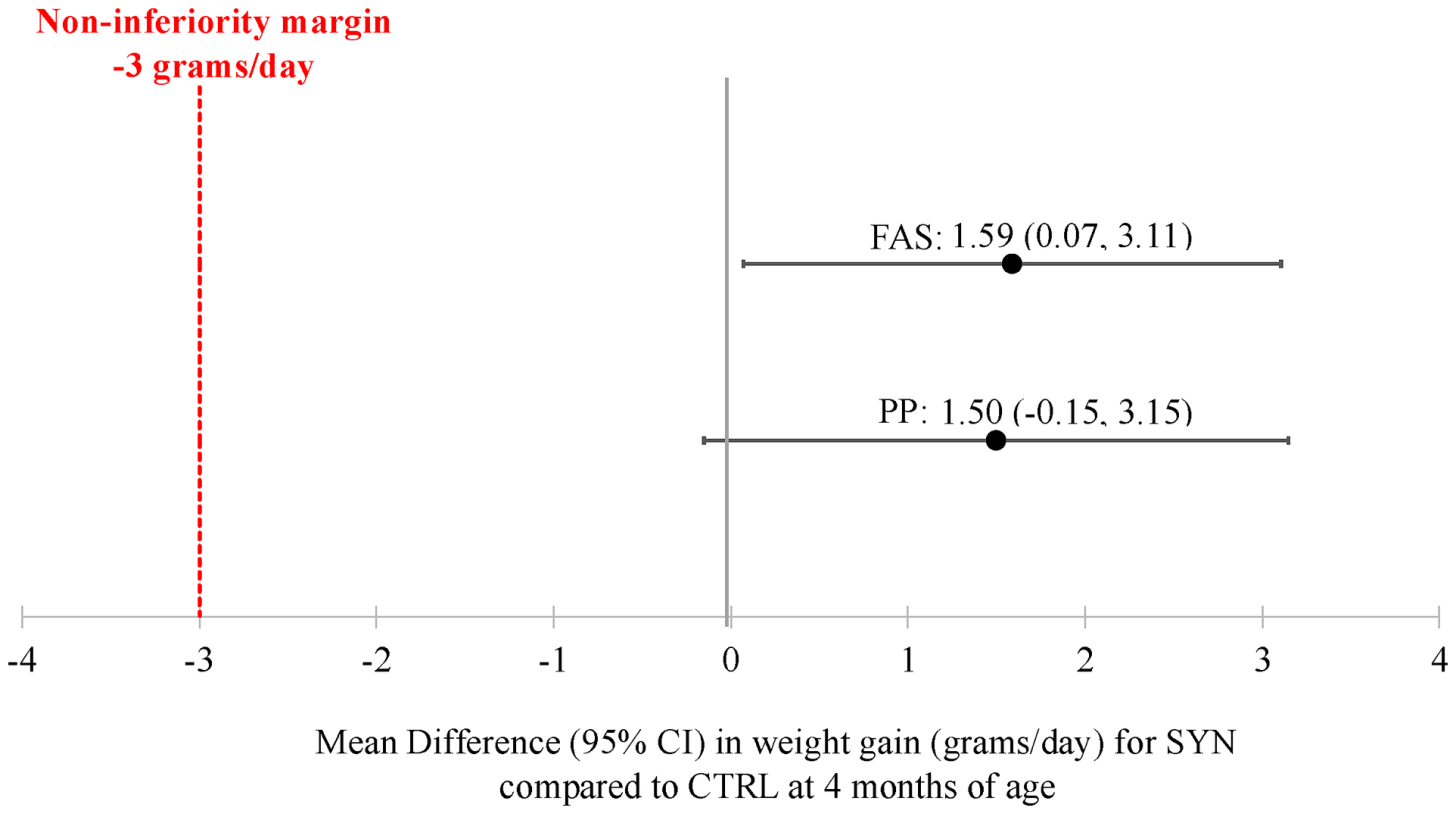
Weight gain velocity through 4 months for the full analysis and per-protocol sets. CI, confidence interval; CTRL, control formula group; FAS, full analysis set; PP, per-protocol population; SYN, experimental formula group. Analyses were performed using ANCOVA, correcting for baseline weight, sex, mode of delivery, and study center. The mean difference was calculated as SYN minus CTRL. Non-inferiority of weight gain velocity for infants in the SYN group compared to the CTRL group was accepted if the lower bound of the two-sided 95% CI on the model-based treatment difference was above the non-inferiority margin of -3 g/day (*p* < 0.001 for both FAS and PP).

Weight gain velocity in the FAS from baseline to 4 months was 29.5 ± 5.9 and 27.9 ± 5.3 g/day for the SYN group (*n* = 95) and CTRL (*n* = 98) groups, respectively. The non-inferiority of weight gain velocity was demonstrated in both the FAS and PP populations, with mean differences (95% CIs) of 1.59 (0.07, 3.11) and 1.50 (−0.15, 3.15), respectively (*p* < 0.001 for both; Figure 2). Increases in length and HC are provided in Supplementary Table S2. Length gain (mm/week) from baseline to 4 months was significantly higher in the SYN (*p* < 0.001) and CTRL (*p* = 0.017) groups compared to the BF group. Similarly, infants in the SYN (*p* = 0.001) and CTRL (*p* < 0.001) groups had greater HC gains compared to BF infants.

**Figure 3:**
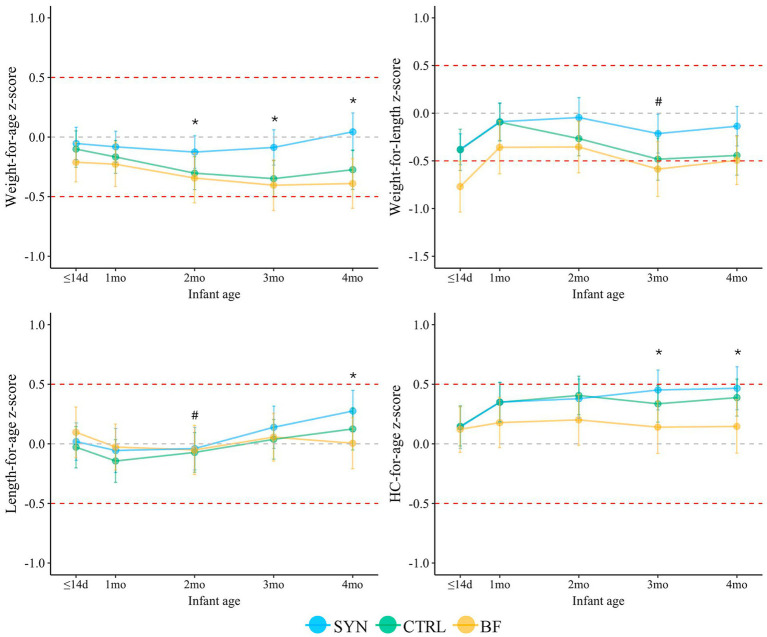
Infant anthropometric z-scores through 4 months for the full analysis set. BF, breastfed group; CTRL, control formula-fed group; HC, head circumference; SYN, experimental formula-fed group. Analyses were performed using propensity score-adjusted ANCOVA, correcting for baseline value, sex, mode of delivery, and study center. *SYN and CTRL were significantly different from BF (*p* < 0.05). #SYN was significantly different from BF (*p* < 0.05). *p*-values were adjusted for multiple comparisons using Benjamini-Hochberg correction.

Significant differences were also observed across the SYN, CTRL, and BF infants in the FAS population in corresponding z-scores for weight-for-age, weight-for-length, length-for-age, and HC-for-age (Figure 3). Both SYN (*p* < 0.001) and CTRL (*p* = 0.016) infants grew slightly faster than BF infants according to the weight-for-age z-score comparisons at 4 months. Infants in the SYN group also had significantly higher weight-for-length z-scores compared to BF at 3 months (*p* = 0.046), with a trend toward higher scores at 4 months (*p* = 0.053), whereas CTRL and BF infants showed similar outcomes (*p* = 0.272). HC-for-age z-scores were also higher at 4 months in the SYN (*p* = 0.002) and CTRL groups (*p* < 0.001) compared to BF infants. Similarly, length-for-age z-scores at 4 months were higher in the SYN (*p* < 0.001) and CTRL (*p* = 0.027) groups compared to the BF group. Despite these differences across groups, all infants were within normal range, with the mean (95% CI) values tracking closely with the WHO median through 4 months.

### 3.4 GI tolerance and stool patterns

**Figure 4:**
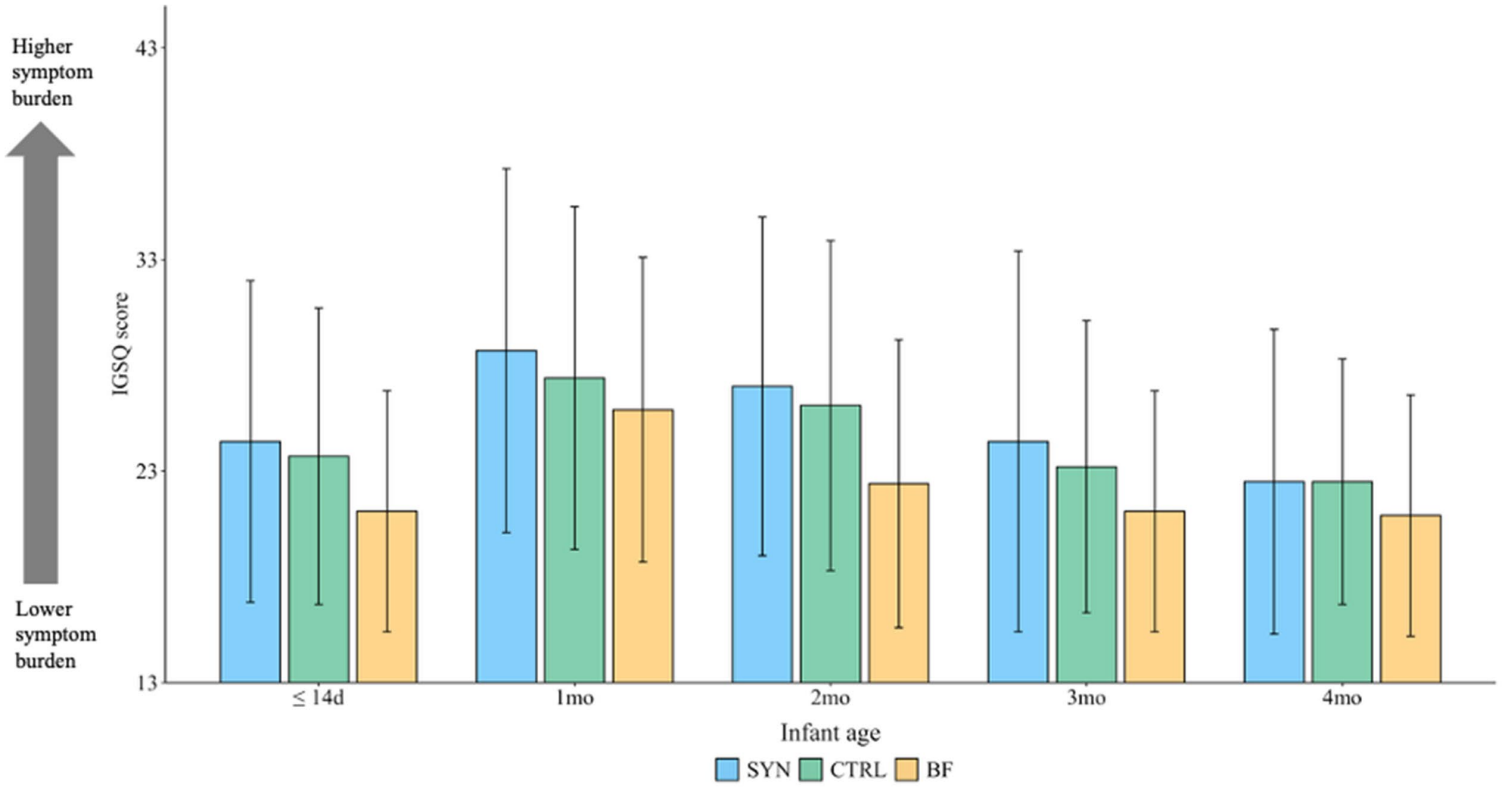
Mean Infant Gastrointestinal Symptom Questionnaire (IGSQ) scores through 4 months for the full analysis set. BF, breastfed group; CTRL, control formula-fed group; SYN, experimental formula-fed group. The IGSQ index score is calculated from the IGSQ questionnaire and ranges from 13 to 65. Scores from 13 to 23 indicate good GI tolerance, scores >23 to 30 suggest some GI distress, and scores >30 to 65 indicate clinically meaningful GI discomfort. Values are presented as mean ± SD and were analyzed using propensity score-adjusted ANCOVA, correcting for baseline value, age, and study center. *p*-values were adjusted for multiple comparisons using Benjamini-Hochberg correction. There were no significant differences between groups at any time point (all *p* > 0.05).

IGSQ index scores were not significantly different across the three groups from 1 to 4 months in the FAS and overall indicated good tolerance (Figure 4). At baseline, the mean±SD scores were 24.4 ± 7.6, 23.7 ± 7.0, and 21.1 ± 5.7 for the SYN, CTRL, and BF groups, respectively, while at 4 months, the mean scores were 22.5 ± 7.2, 22.5 ± 5.8, and 20.9 ± 5.7, respectively. There were no significant differences in individual domain scores for crying, fussiness, flatulence, and spitting up/vomiting observed across the three groups. However, higher domain scores for stooling were noted for SYN infants (4.0 ± 1.9) compared to CTRL (3.4 ± 1.9; *p* = 0.011) and BF (2.8 ± 1.5; *p* = 0.005) infants at 1 month; this difference was not significant at other timepoints.

Stool patterns are presented in Supplementary Table S3. Mean stool frequency was lower at each visit from 1 month to 4 months in the SYN group compared to the BF group and from 2 to 4 months in CTRL compared to BF (all *p* < 0.05). Stool consistency was similar in both the SYN and CTRL groups (closer to “loose”), while the BF group was closer to “watery” at one month (*p* = 0.037) and in only the SYN group at 3 months (*p* = 0.044). Scores moved closer to “formed” in all groups by 4 months. The number of days with difficulty passing stools was similar across the three feeding groups, except at 3 months, when both the SYN (24; *p =* 0.028) and CTRL (20; *p =* 0.030) groups showed higher numbers of days with difficulty passing stools compared with the BF infants. No significant differences in stool frequency, consistency, or difficulty passing stools were observed between SYN and CTRL infants at any time point.

### 3.5 Adverse events and medication use

Physician-reported AEs were similar between the SYN, CTRL, and BF infants (Table 3). A total of 100 SYN, 91 CTRL, and 54 BF infants had at least one AE. However, these differences were not statistically significant. There were 18, 15, and 6 infants with an SAE in the SYN, CTRL, and BF groups, respectively. The majority of serious AEs (39 out of 43 events) were categorized in the infections and infestations system organ class (SOC), and most AEs of interest were upper or lower respiratory tract infections. No significant differences in severity were observed, with the majority of AEs categorized as mild. In the SYN group, 40 AEs (28 infants) were considered probably related, and one AE was deemed related to the study product (cow’s milk protein allergy); 16 infants discontinued the study. There were 45 AEs (30 infants) in the CTRL group, all considered probably related to the study product; 14 infants discontinued the study. Four infants in the BF group discontinued the study. There were no notable or significant differences in the incidence of AEs among the SYN, CTRL, and BF groups by SOC.

**Table 3:** Physician-reported adverse events through 4 months for the safety analysis set^a^.

Variable	SYN *n* = 118		CTRL *n* = 115		BF *n* = 81	
	Events n	Infants n (%)	Events n	Infants n (%)	Events n	Infants n (%)
Any AE	331	100 (85)	288	91 (79)	139	55 (68)
Serious AE	18	18 (15)	17	15 (13)	8	6 (7)
Severity						
Mild	265	87 (74)	235	77 (67)	114	51 (63)
Moderate	55	35 (30)	41	25 (22)	21	11 (14)
Severe	11	8 (7)	12	10 (9)	4	3 (4)
Relation to the study formula						
Probable	40	28 (24)	45	30 (26)	–	
Related	1	1 (1)	0	0		
Unlikely	65	31 (26)	78	33 (29)		
Unrelated	225	82 (70)	165	73 (63)		
Reason for study discontinuation						
Yes	24	16 (14)	26	14 (12)	4	3 (4)

a AE, adverse event; BF, breastfed group; CTRL, control formula-fed group; SYN, experimental formula-fed group. There were no significant differences among the groups (all *p* > 0.05). All *p*-values were adjusted for multiple comparisons using the Benjamini-Hochberg correction.

### 3.6 Fecal bifidobacteria abundance

Fecal samples were available from most infants, with samples available from 100, 95, and 68 infants in the SYN, CTRL, and BF groups at baseline, respectively, and 83, 78, and 53 infants in these groups at 3 months, respectively.

**Figure 5:**
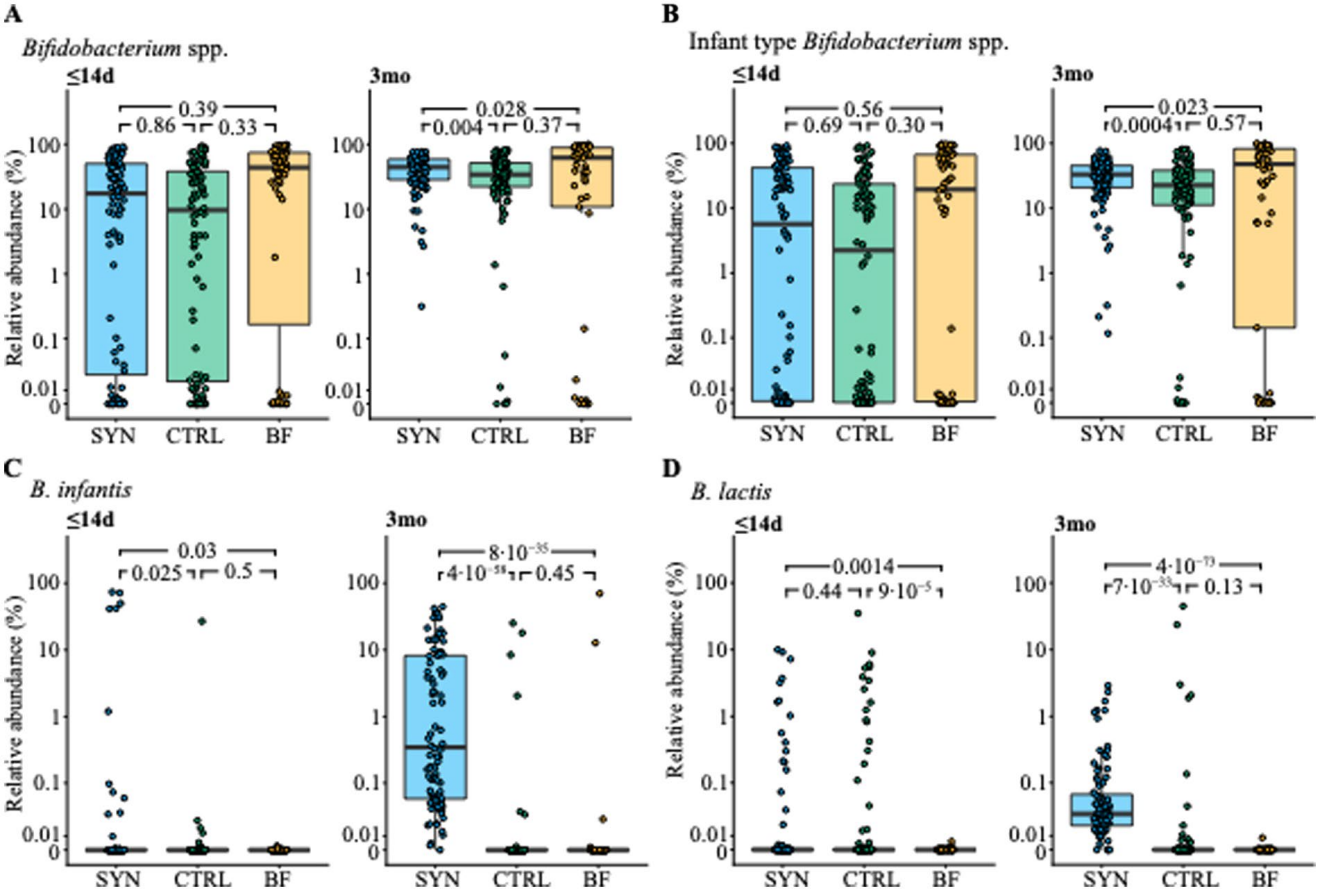
Abundance of **(A)** *Bifidobacterium* species, **(B)** infant-type *Bifidobacterium* species, **(C)** *B. infantis* and **(D)** *B. lactis* at baseline (≤14 days [d]) and 3 months (3mo) for the full-analysis set. BF, breastfed group; CTRL, control formula-fed group; SYN, experimental formula-fed group. Box and whisker plots are shown with individual subjects plotted as circles. Statistical significance between groups is indicated by *p*-values from a cross-sectional, bias-corrected mixed-effects model, which corrects for baseline age and study center.

At baseline, no difference in the abundance of bifidobacteria was observed between the different feeding groups. At 3 months, the SYN group showed a significantly higher relative abundance of bifidobacteria compared to CTRL infants (*p* = 0.004; Figure 5A). BF infants showed the highest mean abundance, although some BF infants exhibited very low abundance, resulting in high variability across the group, similar to what was observed in the CTRL group. In contrast, the SYN group did not exhibit such variability in abundance at 3 months. A similar higher abundance and reduced variability toward the lower end was observed for infant-type bifidobacteria (mainly comprised of *B. longum* subsp. *infantis*, *B. bifidum*, *B. breve*, and *B. longum* subsp. *longum*) in 3-month-old SYN infants compared to CTRL infants (Figure 5B). The highest median abundance of infant-type bifidobacteria was observed in BF infants. These results were also observed in the PP and sub-PP populations (both *p* ≤ 0.003; Supplementary Figure S2). The significantly altered bifidobacteria abundances between the CTRL and SYN groups are shown in Supplementary Figure S3. The individual *Bifidobacterium* species *B. lactis* and *B. infantis,* which correspond to the two provided probiotic species, were highest in the SYN group at 3 months compared to both CTRL and BF groups (Figures 5C,D).

### 3.7 Probiotic *B. infantis* LMG11588 tracking

**Figure 6:**
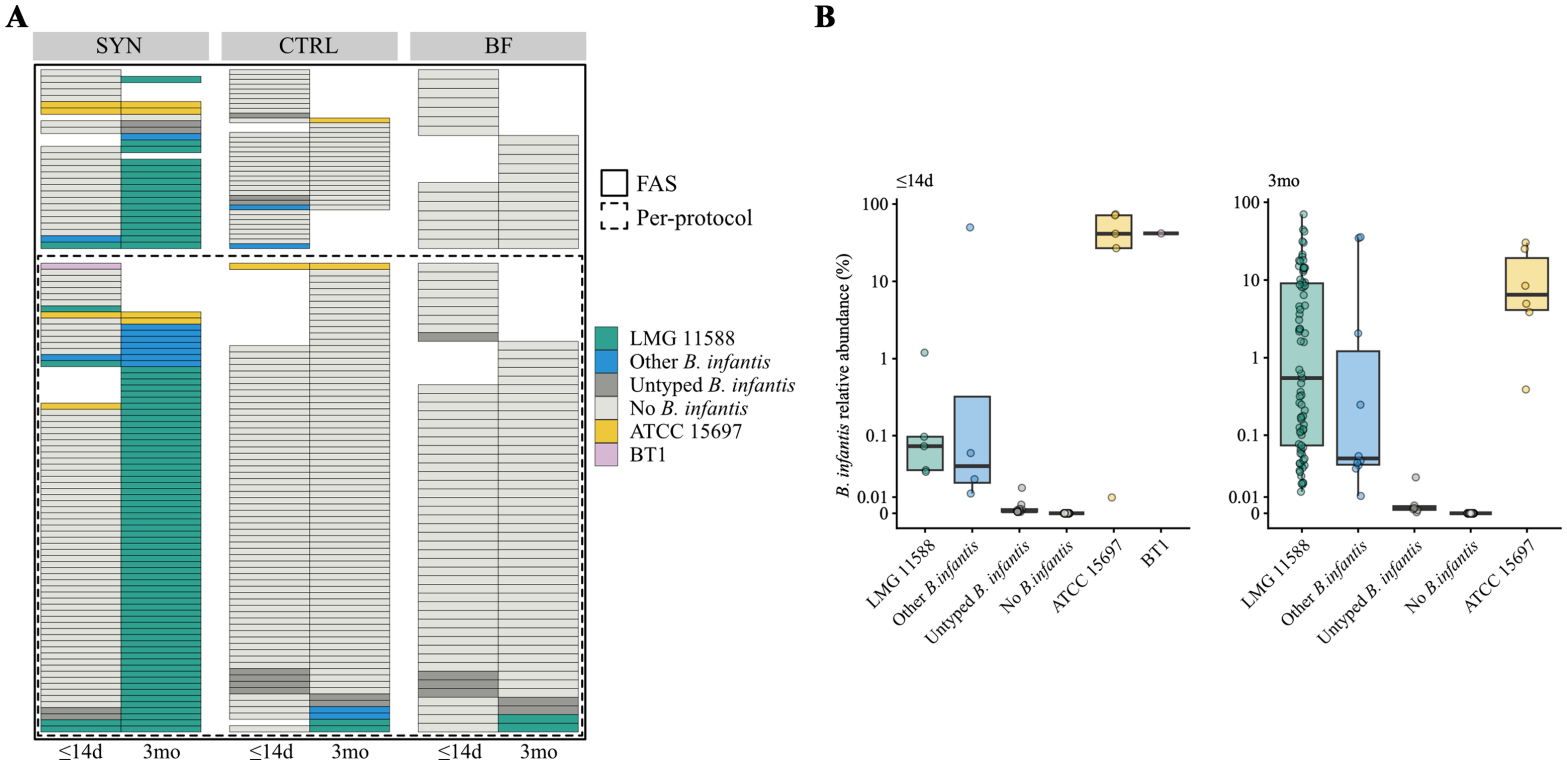
*B. infantis* strain tracking at baseline (≤14 days [d]) and 3 months (3mo) for the full analysis and per-protocol sets. **(A)** *B. infantis* strain categorization based on SNV-level variation shown for each infant (horizontal rows) in the per-protocol and full analysis set at each visit, stratified by intervention group. The white area indicates unavailable samples. Infants in the per-protocol analysis are grouped. **(B)** Relative abundance of *B. infantis* stratified by *B. infantis* strain categorization and visit across all three study groups in the full analysis set. BF, breastfed group; CTRL, control formula-fed group; SYN, experimental formula-fed group.

At baseline, very few infants—regardless of feeding group—had detectable and typable *B. infantis* in their feces. At 3 months, all but one SYN infant had detectable *B. infantis* in their feces, with the majority of infants (83.7%) harboring the probiotic strain LMG11588 (Figure 6A). Approximately 16.3% of SYN infants showed untyped or other *B. infantis* strains at 3 months, while in both the CTRL and BF groups, fewer infants had detectable *B. infantis*. In the SYN group, very few infants showed the strain LMG11588 at baseline, and very few infants in the CTRL and BF groups had detectable *B. infantis* LMG11588 at 3 months of age. At baseline, *B. infantis* strains ATCC 15687 and BT1 were present at a much higher abundance compared to other detected strains. At 3 months, the abundance of *B. infantis* strain LMG11588 ranged from just above 0.01% to approximately 90%, with half of the infants having LMG11588 between 0.1 and 10% of all measured microbes. Other typeable strains were similarly abundant (Figure 6B).

### 3.8 Fecal pathogenic bacteria and other microbes

**Figure 7:**
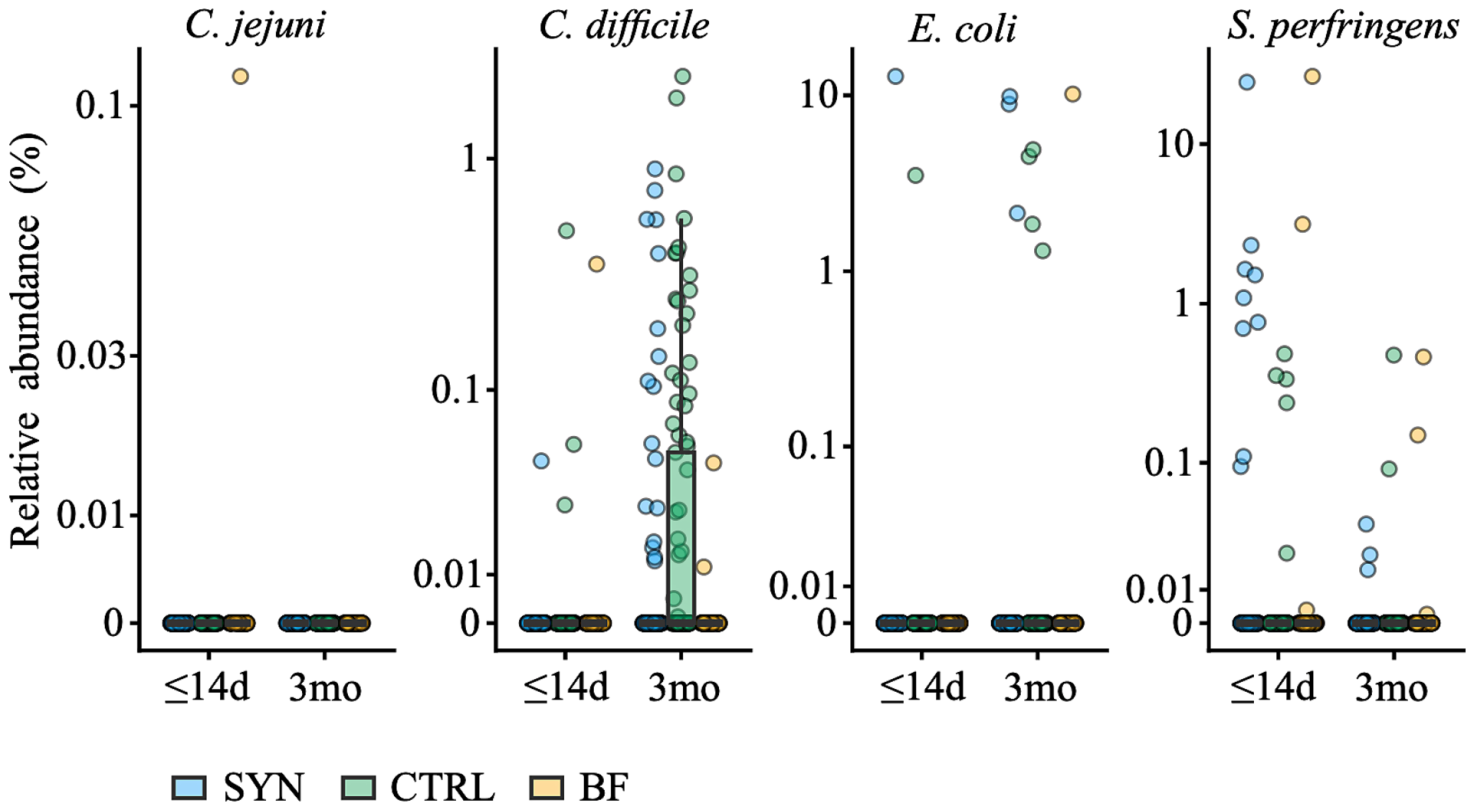
Abundance of potentially pathogenic species at baseline (≤14 days [d]) and 3 months (3mo) for the full analysis set. BF, breastfed group; CTRL, control formula-fed group; SYN, experimental formula-fed group. The following species were detected: *Campylobacter jejuni* (*C. jejuni*), toxigenic *Clostridiodes difficile* (*C. difficile*), pathogenic *Escherichia coli* (*E. coli*), and *Sarcina perfringens* (*S. perfringens;* formerly *Clostridium perfringens*).

At baseline, potentially pathogenic bacteria were detected in very few infant fecal samples. At 3 months, mainly toxigenic *Clostridioides difficile* emerged, showing a significantly higher prevalence and abundance in CTRL compared to the SYN group (*p = 0.0193*; *p = 0.00521*, respectively) and the BF group (*p* < 0.001 for both; Figure 7). The taxa showing a significantly different prevalence and abundance at baseline and 3 months between the SYN and CTRL groups are shown in Supplementary Figure S3. Notably, few taxa differed between the SYN and CTRL groups after false discovery correction. Among these, the prevalence and abundance of the mucolytic bacterium *Ruminococcus gnavus* were lower in the SYN group compared to the CTRL group at 3 months of age. They were lowest in prevalence and abundance in BF.

### 3.9 Fecal pH and organic acids

**Figure 8:**
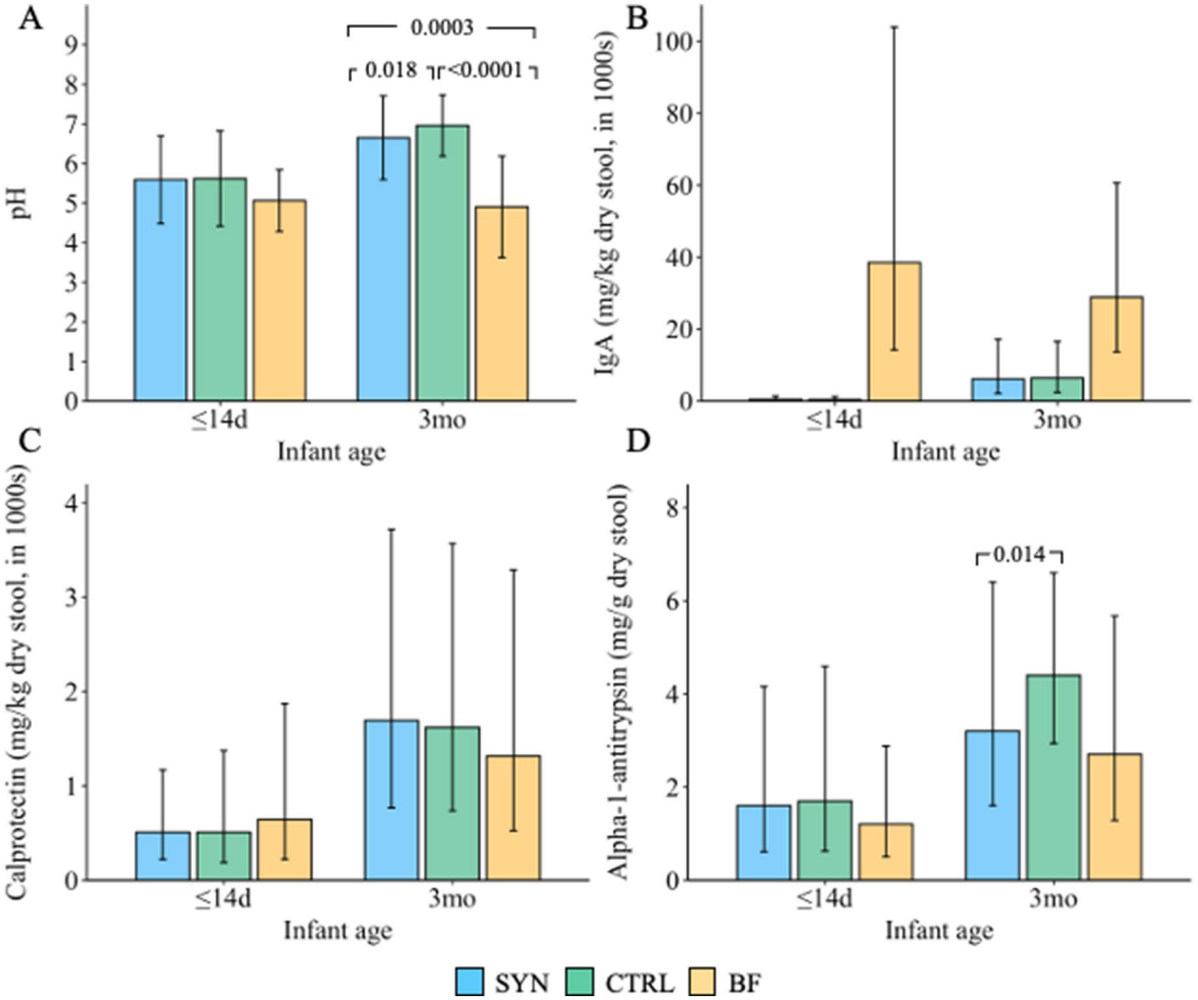
Fecal **(A)** pH (presented as mean ± SD); values below the lower limit of quantification [LLOQ] were imputed by LLOQ/2, and values above the upper limit of quantification [ULOQ] were imputed by ULOQ; **(B)** IgA; **(C)** calprotectin; and **(D)** alpha-1 antitrypsin (all presented as geometric mean and geometric SD) at baseline (≤14 days [d]) and 3 months (3mo). BF, breastfed group; CTRL, control formula-fed group; SYN, experimental formula-fed group. All outcomes were analyzed using propensity score-adjusted ANCOVA, correcting for baseline value, age, and study center. *p*-values were adjusted for multiple comparisons using Benjamini-Hochberg correction.

Significant differences in fecal pH (Figure 8A) and some SCFAs (Supplementary Table S4) were observed at 3 months. Fecal pH was significantly lower in the SYN group compared to CTRL infants (*p* = 0.018), but levels were higher in both formula-fed groups compared to BF infants (both *p* ≤ 0.0003). The relative proportion of acetic acid was significantly higher in the BF group compared to the SYN or CTRL groups (SYN vs. BF: *p* = 0.010; CTRL vs. BF: *p* = 0.032). Compared to BF infants, a higher proportion of propionic acid was found in the SYN group (*p* = 0.005), but there were no differences between the CTRL and BF groups. In contrast, proportions of butyric and valeric acid were not significantly different across the three groups. Similarly, at 3 months, DL-lactic acid levels were similar in the SYN and CTRL groups and significantly lower compared to the BF group (*p = 0.002* for both).

### 3.10 Fecal markers of immune and gut health

Immune and gut health markers are presented in Figure 8. Levels of sIgA (Figure 8B), calprotectin (Figure 8C), and lipocalin-2 (data not shown) in the SYN and CTRL groups were not significantly different from those of BF infants at 3 months. AAT levels (Figure 8D) were more comparable between the SYN and BF groups; however, AAT was significantly lower in the SYN group compared to the CTRL group (*p* = 0.014). In the BF group, AAT levels were also observed to be lower compared to those in the CTRL group, although this difference did not reach statistical significance (*p* = 0.067).

## 4 Discussion

This is the first study to provide data on growth, GI tolerance, and gut microbiome outcomes in response to feeding with a unique infant formula consisting of a synbiotic blend of 6 HMOs and *B. infantis* plus *B. lactis* across various centers in Europe. The results demonstrate that this study formula supported healthy, age-appropriate growth and was safe and well-tolerated through 4 months of age. Moreover, the synbiotic formula promoted the expansion of beneficial bifidobacteria, especially infant-type species, in all infants, suggesting the formula could contribute toward minimizing the observed gaps in early microbiome development between formula-fed and BF infants.

Weight gain at 4 months in the SYN group was non-inferior to that of CTRL infants. Although growth anthropometry values were within normal ranges for infants across all three study groups and closely tracked the WHO median at 4 months, the SYN group had higher weight-for-age, weight-for-length, height-for-age, and length-for-age z-scores compared to the BF infants. Despite these differences, both formula-fed groups exhibited normal, age-appropriate growth. Recently, a systematic review that evaluated growth outcomes associated with manufactured HMO supplementation at different concentrations and in diverse populations reported similar age-appropriate growth and no significant differences between intervention groups and controls (6). Here, we show that this is also the case when HMOs are combined with a specific probiotic combination of *B. infantis* LMG11588 and *B. lactis* CNCM I-3446.

Good GI tolerance was demonstrated across all groups, and no significant differences were observed when examined using the IGSQ composite score. Stool consistency and difficulty in passing stools were similar across all groups, but stool frequency was slightly lower in the formula-fed infants compared to the BF infants at 4 months. These findings are generally consistent with an earlier trial conducted in a similar population of infants, which examined the effects of a probiotic-containing infant formula supplemented with 2’FL from birth to 4 months of age; the trial reported comparable gastrointestinal symptoms and stool patterns between the formula-fed groups (7). Similarly, a recent systematic review that identified studies examining the impact of manufactured HMOs on tolerance reported that HMO supplementation was well-tolerated among infants across the identified studies (6). These data are generally consistent with the good GI tolerance observed in a supplement trial with the probiotic *B. infantis* LMG11588 (17).

The study results also demonstrated the overall expansion of beneficial bifidobacteria, specifically infant-type species, which encompass species capable of metabolizing specific HMOs and producing several compounds (e.g., organic acids, phenylalanine, and tryptophan derivatives) identified to modulate immune system development (20). The initial years of life are a critical period for development; bifidobacteria rapidly colonize the gut following birth and remain plentiful in the intestinal microbiota throughout life (21–23). Importantly, high *bifidobacteria* abundance in breastfed infants during the first months is achieved in the presence of commensal bifidobacteria able to metabolize HMOs (10). It is therefore meaningful that this formula resulted in similar bifidobacteria levels among SYN and BF infants (24). Similar to the BF group, SYN formula-fed infants had a significantly lower presence and abundance of opportunistic pathogens, mainly toxigenic *C. difficile*. A similar observation was previously reported in infants fed a formula supplemented with a blend of 5HMO without any probiotics (9), indicating that this effect is likely due to the supplemented HMOs rather than the probiotics. Although not yet fully established, the altered compositional and functional gut ecology, characterized by higher abundance of bifidobacteria and lower fecal pH, may explain the reduced prevalence of toxigenic *C. difficile* (25). Mechanistically, acetic acid produced through bifidobacterial metabolism may also support gut barrier function, as recently shown in mechanistic models (26).

Interestingly, the commensal infant-type bifidobacteria *B. infantis* were only rarely observed in BF and CTRL infants compared to SYN infants through 3 months. A similar low prevalence was recently reported in other geographies (27), which is consistent with the general loss of *B. infantis* and the reduced capacity for HMO metabolism observed in industrialized settings compared to developing countries (12). Upon supplementation with the probiotic *B. infantis* LMG11588, a significant increase in the prevalence and abundance of the species *B. infantis* was achieved, and essentially all infants in the SYN group showed the presence of *B. infantis*. Strain tracking confirmed that the probiotic LMG11588 was mostly present, with a few other *B. infantis* strains also detected. Of the latter, few were identifiable as known strains. From the collected information on the given concomitant supplements, we could not determine whether some of the identified *B. infantis* strains were introduced through the consumption of an unauthorized probiotic supplement; however, this may explain the observed effect.

Additionally, differences in pH and some SCFAs were observed as general indicators of gut ecology. Primarily, the reduced fecal pH seen in the SYN group compared to the CTRL group is an indicator of microbial activity, indicating higher microbial activity in the SYN group. Fecal markers of gut health (AAT [a measure of gut barrier integrity], calprotectin [a measure of gut inflammation]) and immune development (sIgA) were largely similar in formula-fed infants. For sIgA, the higher amount detected in fecal samples from BF infants’ feces is mostly from breastmilk, while in formula-fed infants, the measured amounts reflect the sIgA produced after birth in the infant’s gut. Similar to a previous multi-center trial studying the impact of an infant formula containing a blend of 5 HMOs (9), the current trial also observed reduced AAT levels in the feces of the SYN group compared to the CTRL group, and this was more similar to the observed amounts in BF infants. Collectively, the lower pH and AAT values indicate that SYN has positive effects on markers of gut health.

Several mechanisms have been proposed regarding the pathways by which the various ingredients of this formula may affect infant gut and immune outcomes. HMOs have numerous direct and indirect functions through interactions with epithelial cells, pathogens, and metabolites within and beyond the gut, making notable contributions to bifidobacteria colonization during early infancy (28). Additionally, HMOs have been linked to metabolic pathways that affect gut SCFAs and pH levels through the metabolic activity of gut bacterial communities (6). The gut also includes bifidobacteria, which interact with immune cells and affect immunomodulatory functions (9, 28), ferment indigestible glycans (28), interact with bile in the gut (28), and are known to metabolize HMOs (9, 28). The interactions between HMOs and bifidobacteria could further promote a healthy gut environment and immune development by creating a synbiotic effect, and this is especially important given current evidence suggesting that not all infants’ gut microbiomes metabolize HMOs equally (10, 20, 29). A recent study examining the effect of *B. infantis* LMG11588 combined with HMOs in *ex vivo* colonic incubation bioreactors seeded with fecal background microbiota from infant and toddler donors showed improvements in HMO metabolism for all donors, characterized by increases in SCFAs (11). While further studies are necessary to better describe these mechanisms, existing knowledge suggests that the ingredients in this formula synbiotically alter the gut environment to more closely resemble the profile of BF infants.

Strengths of this study include the randomized design and the use of a propensity score to include the BF reference group in analyses, despite this being a non-randomized group. Additionally, a high number of infants completed the study from enrollment through 4 months, which minimizes the potential for bias from missing data and losses to follow-up. This study was also conducted in centers across multiple European countries, thereby providing a more representative sample of infants. Validated tools and laboratory methodologies were utilized to assess the outcomes of interest. Examining fecal biomarkers and the microbiota also strengthened the evaluation of this synbiotic formula’s impact on infant immunity and gut development. Lastly, the unique blend of manufactured HMOs in this formula is an innovative concept and could have a beneficial impact on the long-term health trajectories of infants receiving formula. Limitations of this study include the relatively high proportion of enrolled infants with major protocol deviations (29%) and the inability to complement the results and their interpretation with fecal cytokine results, as more than 90% of values fell below the level of quantification.

## 5 Conclusion

In conclusion, this partially hydrolyzed infant formula, which includes a specific synbiotic blend of 6 HMOs and the probiotics *B. infantis* LMG11588 and *B. lactis* CNCM I-3446, was safe and well-tolerated up to 4 months of age. Breastfeeding is the ideal source of nutrition for infants. Given the importance of breastfeeding for infants to have a healthy, long-term developmental trajectory, it is crucial to maximize the benefits that formula can provide in cases where breastfeeding is not possible. This study formula supported appropriate infant growth and increased the relative abundance of bifidobacteria, thereby positively influencing gut health. Additional findings through 15 months of age will provide a longitudinal perspective of the impact of the formula on these endpoints.

## Data Availability

The raw data supporting the conclusions of this article will be made available by the authors without undue reservation.

## Glossary

2’FL: 2’fucosyllactose
3-FL: 3-fucosyllactose
3’SL: 3’sialyllactose
6’SL: 6’sialyllactose
AAT: Alpha-1 antitrypsin
AE: Adverse event
ANCOVA: Analysis of covariance
BF: Breastfed
BMI: Body mass index
CTRL: Control formula
CFU: Colony forming unit
CI: Confidence interval
CONSORT: Consolidated Standards of Reporting Trials
DFL: 2′,3-di-O-fucosyllactose
DNA: Deoxyribonucleic acid
eCRF: Electronic case report form
SYN: Experimental formula
ELISA: Enzyme-linked immunosorbent assay
FAS: Full analysis set
FUF: Follow-up formula
GI: Gastrointestinal
GUM: Growing-up milk/formula
HC: Head circumference
IF: Starter infant formula
IGSQ: Infant Gastrointestinal Symptom Questionnaire
IID: Infant illness diary
ITT: Intention-to-treat
LNT: Lacto-N-tetraose
NGS: Next generation sequencing
PP: Per-protocol
SAE: Serious adverse event
sIgA: Secretory immunoglobulin A
SD: Standard deviation
SOC: System organ class
SAS: Safety analysis set
Sub-PP: Sub-per-protocol
V0: Baseline study visit
V1: Study visit at 1 month of age
V2: Study visit at 2 months of age
V3: Study visit at 3 months of age
V4: Study visit at 4 months of age
V5: Study visit at 6 months of age
V6: Study visit at 9 months of age
V7: Study visit at 12 months of age
V8: Study visit at 15 months of age
