# Correction: Partially hydrolyzed, whey-based infant formula with six human milk oligosaccharides, *B. infantis* LMG11588, and *B. lactis* CNCM I-3446 is safe, well tolerated, and improves gut health: a staged analysis of a randomized trial

**DOI:** 10.3389/fnut.2025.1676657

**Published:** 2025-09-12

**Authors:** Jean-Charles Picaud, Olivier Claris, Mercedes Gil-Campos, Ignacio Salamanca De La Cueva, Luc Cornette, Philippe Alliet, André Léké, Mireille Castanet, Hugues Piloquet, Virginie de Halleux, Delphine Mitanchez, Yvan Vandenplas, Pierre Maton, Frank Jochum, Dirk Olbertz, Sergio Negre Policarpo, Luca Lavalle, Cecilia Fumero, Paula Rodriguez-Garcia, Janne Marie Moll, Irma Silva-Zolezzi, Boutaina Zemrani, Nicholas P. Hays, Norbert Sprenger, Javier Miranda-Mallea

**Affiliations:** ^1^Department of Neonatology, Hôpital de La Croix-Rousse, Lyon, France; ^2^CarMen Laboratory, INSERM, INRA, Université Claude Bernard Lyon 1, Pierre-Bénite, Lyon, France; ^3^Department of Neonatology, Hôpital Femme Mère Enfants, Bron, France; ^4^Paediatric Metabolism Unit, Reina Sofia University Hospital, University of Córdoba, IMIBIC, CIBEROBN, Córdoba, Spain; ^5^Department of Pediatrics, Instituto Hispalense de Pediatria, Sevilla, Spain; ^6^Department of Neonatology, AZ Sint-Jan Hospital, Brugge, Belgium; ^7^Department of Pediatrics, Jessa Hospital, Hasselt, Belgium; ^8^Neonatal Medicine and Intensive Care, Centre Hospitalier Universitaire Amiens Picardie, Amiens, France; ^9^CIC INSERM U1404, Department of Pediatrics, Rouen University Hospital Charles Nicole, Rouen, France; ^10^Child Chronic Disease Service, Centre Hospitalier Universitaire de Nantes, Nantes, France; ^11^Neonatology Division, Centre Hospitalier Universitaire de Liège – Centre Hospitalier Universitaire de la Citadelle, Liège, Belgium; ^12^Service de Néonatologie, Centre Hospitalier Universitaire de Tours, Tours, France; ^13^KidZ Health Castle, Vrije Universiteit Brussel, UZ Brussel, Brussels, Belgium; ^14^Service Néonatal, Clinique CHC-Montlégia, Liège, Belgium; ^15^Department of Pediatrics, Evangelisches Waldkrankenhaus Spandau, Berlin, Germany; ^16^Department of Neonatology, Klinikum Südstadt Rostock, Rostock, Germany; ^17^Department of Pediatrics, Hospital Quironsalud, Valencia, Spain; ^18^Clinical Research Unit, Nestlé Research, Lausanne, Switzerland; ^19^Cmbio, Copenhagen, Denmark; ^20^Clinical and Nutritional Research Unit, Nestlé Product Technology Center – Nutrition, Vevey, Switzerland; ^21^Nestlé Institute of Health Sciences, Nestlé Research, Lausanne, Switzerland; ^22^Department of Pediatrics, Hospital Vithas, Valencia, Spain

**Keywords:** bifidobacteria, gastrointestinal tolerance, growth, gut health, microbiota

In the published article, there was an error in the Supplementary materials. Supplementary Tables S2, S3, and S4 were omitted. In addition, Supplementary Figure S1, Supplementary Figure S2, and Supplementary Figure S3 were published without figure legends with the original version of this paper. To correct these errors, the files have been replaced with a consolidated file containing all the Supplementary Material in one document.

The original version of this article has been updated.

