# Supplementary material for: Partially hydrolyzed, whey-based infant formula with six human milk oligosaccharides, B. infantis LMG11588, and B. lactis CNCM I-3446 is safe, well tolerated, and improves gut health: a staged analysis of a randomized trial
Source: Front Nutr. 2025 Jul 23;12:1628847. doi: 10.3389/fnut.2025.1628847 (PMC12325064; doi:10.3389/fnut.2025.1628847)
Supplement: Supplementary file 1 [file Data_Sheet_1.PDF]

Supplementary Material

1 Supplementary Figures and Tables

1.1 Supplementary Figures

Figure S1. Graphical representation of study design. The total duration of the study intervention was 15 months consisting of a baseline visit at or before age 14 days, and 8 subsequent in-person visits at ages 1, 2, 3, 4, 6, 9, 12, and 15 months. The study formulas were staged according to the age of the infant (1st age infant formula: 0 up to 6 months; 2nd age follow-up formula: 6 up to 12 months; 3rd age growing-up milk [GUM]: 12 through 15 months). * Two staged statistical analyses were performed after all infants completed the 4- and 12-month visits.

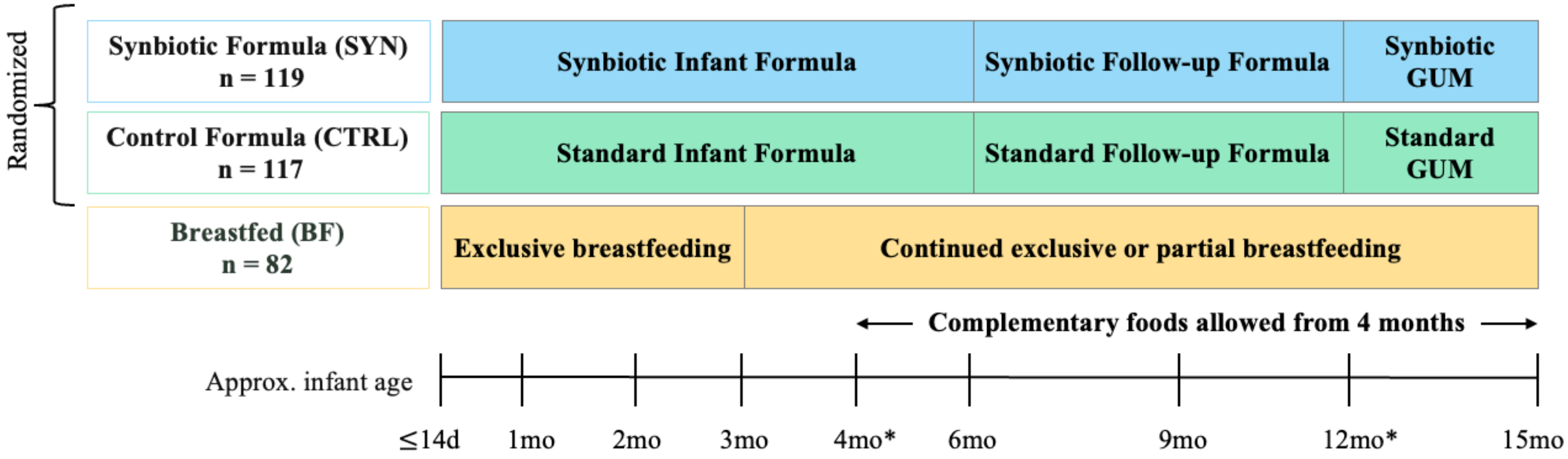

Figure S2. Sensitivity analysis of *Bifidobacteria* abundance at baseline and age 3 months for the (**A**) per-protocol (PP) and (**B**) sub-per-protocol (sub-PP) analysis sets. BF, breastfed group; CTRL, control formula-fed group; SYN, experimental formula-fed group. Models were adjusted for baseline age and study center.

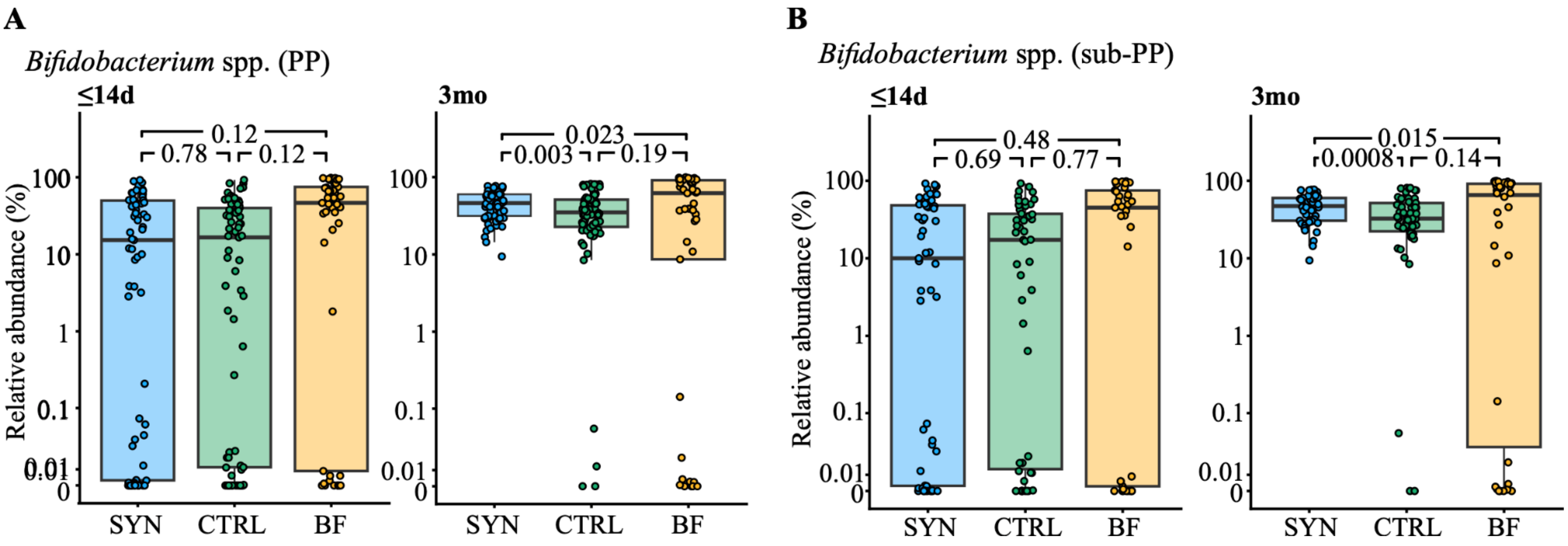

Figure S3. Taxa prevalence and abundance that changed between the SYN and CTRL groups at baseline and age 3 months for the full analysis set. BF, breastfed group; CTRL, control formula-fed group, SYN: experimental formula-fed group. Panels **A** and **B**: Overview of microbiota features significantly differentially prevalent between CTRL and SYN ($p<0.05$; with dot indicating fall discovery rate [FDR] < 0.1) based on a model including SYN, CTRL, and BF in the model fitting. Comparisons are shown stratified on taxonomic level and colored by the intervention group with the highest abundance. Significance was assessed using a linear mixed model. Panels **C** and **D**: Overview of microbiota features significantly differentially abundant between intervention groups ($p<0.05$; with dot indicating FDR < 0.1) based on a model including SYN, CTRL, and BF in the model fitting. Comparisons are shown stratified on taxonomic level and colored by the intervention group with the highest abundance. Significance was assessed using a linear mixed model.

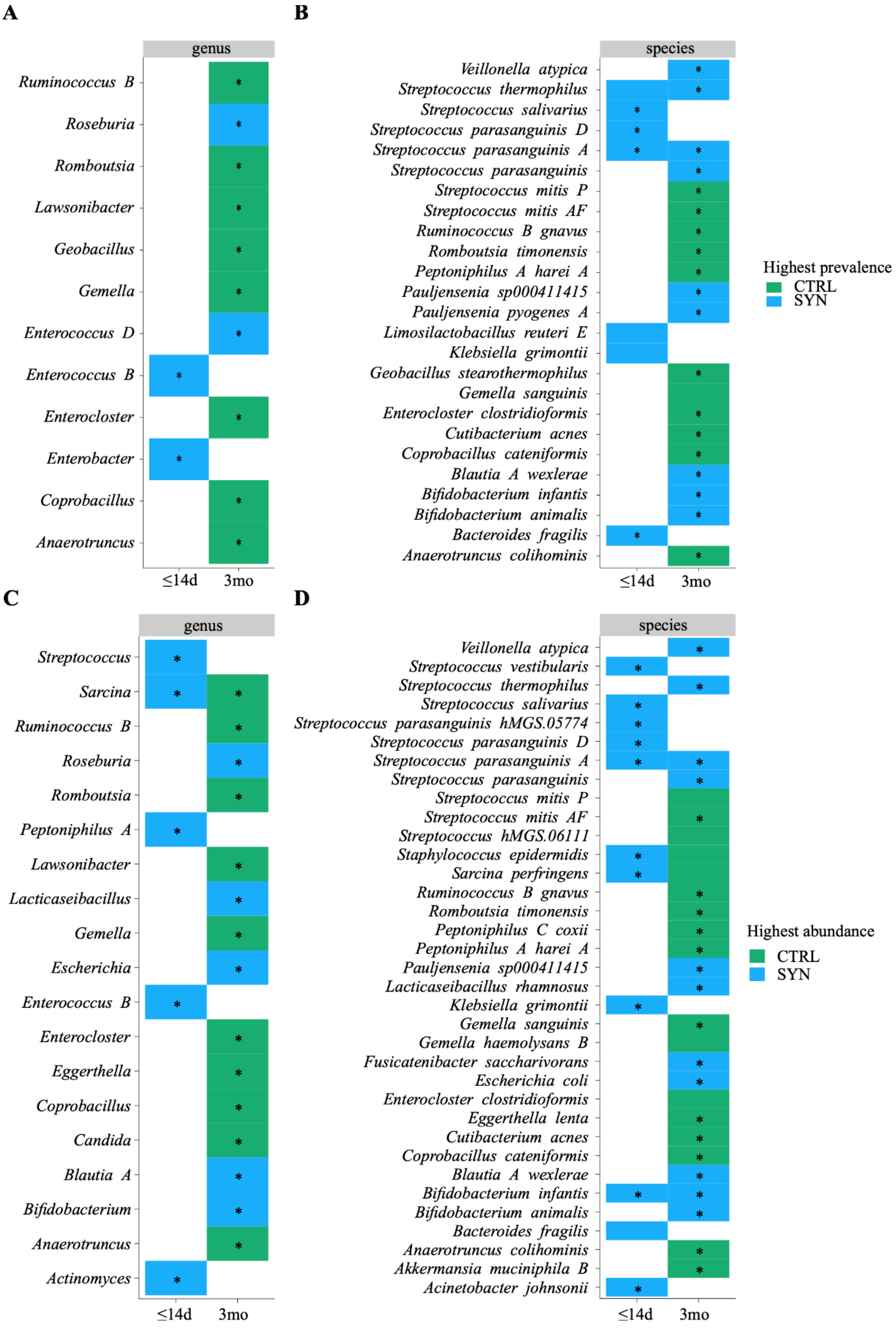
A
genus
Ruminococcus B
Roseburia
Romboutsia
Lawsonibacter
Geobacillus
Gemella
Enterococcus D
Enterococcus B
Enterocloster
Enterobacter
Coprobacillus
Anaerotruncus
≤14d
3mo
B
species
Veillonella atypica
Streptococcus thermophilus
Streptococcus salivarius
Streptococcus parasanguinis D
Streptococcus parasanguinis A
Streptococcus parasanguinis
Streptococcus mitis P
Streptococcus mitis AF
Ruminococcus B gnavus
Romboutsia timonensis
Peptoniphilus A harei A
Pauljensenia sp000411415
Pauljensenia pyogenes A
Limosilactobacillus reuteri E
Klebsiella grimontii
Geobacillus stearothermophilus
Gemella sanguinis
Enterocloster clostridioformis
Cutibacterium acnes
Coprobacillus cateniformis
Blautia A wexlerae
Bifidobacterium infantis
Bifidobacterium animalis
Bacteroides fragilis
Anaerotruncus colihominis
≤14d
3mo
Highest prevalence
CTRL
SYN
C
genus
Streptococcus
Sarcina
Ruminococcus B
Roseburia
Romboutsia
Peptoniphilus A
Lawsonibacter
Lacticaseibacillus
Gemella
Escherichia
Enterococcus B
Enterocloster
Eggerthella
Coprobacillus
Candida
Blautia A
Bifidobacterium
Anaerotruncus
Actinomyces
≤14d
3mo
D
species
Veillonella atypica
Streptococcus vestibularis
Streptococcus thermophilus
Streptococcus salivarius
Streptococcus parasanguinis hMGS.05774
Streptococcus parasanguinis D
Streptococcus parasanguinis A
Streptococcus parasanguinis
Streptococcus mitis P
Streptococcus mitis AF
Streptococcus hMGS.06111
Staphylococcus epidermidis
Sarcina perfringens
Ruminococcus B gnavus
Romboutsia timonensis
Peptoniphilus C coxii
Peptoniphilus A harei A
Pauljensenia sp000411415
Lacticaseibacillus rhamnosus
Klebsiella grimontii
Gemella sanguinis
Gemella haemolysans B
Fusicatenibacter saccharivorans
Escherichia coli
Enterocloster clostridioformis
Eggerthella lenta
Cutibacterium acnes
Coprobacillus cateniformis
Blautia A wexlerae
Bifidobacterium infantis
Bifidobacterium animalis
Bacteroides fragilis
Anaerotruncus colihominis
Akkermansia muciniphila B
Acinetobacter johnsonii
≤14d
3mo
Highest abundance
CTRL
SYN

1.2 Supplementary Tables

Table S1. Consolidated Standards of Reporting Trials (CONSORT) Checklist

Section/Topic	Item No	Checklist item	Reported on page No
**Title and abstract**			
	1a	Identification as a randomised trial in the title	Title page
	1b	Structured summary of trial design, methods, results, and conclusions (for specific guidance see CONSORT for abstracts)	Abstract
**Introduction**			
Background and objectives	2a	Scientific background and explanation of rationale	Section 1
	2b	Specific objectives or hypotheses	Section 1, last paragraph
		**Methods**	
Trial design	3a	Description of trial design (such as parallel, factorial) including allocation ratio	Sections 2.1 & 2.2
	3b	Important changes to methods after trial commencement (such as eligibility criteria), with reasons	Not applicable
Participants	4a	Eligibility criteria for participants	Section 2.1
	4b	Settings and locations where the data were collected	Section 2.1
Interventions	5	The interventions for each group with sufficient details to allow replication, including how and when they were actually administered	Section 2.3 & Fig S1
Outcomes	6a	Completely defined pre-specified primary and secondary outcome measures, including how and when they were assessed	Sections 2.5 through 2.13
	6b	Any changes to trial outcomes after the trial commenced, with reasons	Not applicable
Sample size	7a	How sample size was determined	Section 2.14
	7b	When applicable, explanation of any interim analyses and stopping guidelines	Section 2.1 & Fig S1
Randomisation:			Section 2.2
	8a	Method used to generate the random allocation sequence	

Sequence generation	8b	Type of randomisation; details of any restriction (such as blocking and block size)	Section 2.2
Allocation concealment mechanism	9	Mechanism used to implement the random allocation sequence (such as sequentially numbered containers), describing any steps taken to conceal the sequence until interventions were assigned	Section 2.2
Implementation	10	Who generated the random allocation sequence, who enrolled participants, and who assigned participants to interventions	Section 2.2
Blinding	11a	If done, who was blinded after assignment to interventions (for example, participants, care providers, those assessing outcomes) and how	Section 2.2
	11b	If relevant, description of the similarity of interventions	Section 2.3
Statistical methods	12a	Statistical methods used to compare groups for primary and secondary outcomes	Section 2.15
	12b	Methods for additional analyses, such as subgroup analyses and adjusted analyses	Section 2.15
		**Results**	
Participant flow (a diagram is strongly recommended)	13a	For each group, the numbers of participants who were randomly assigned, received intended treatment, and were analysed for the primary outcome	Figure 1
	13b	For each group, losses and exclusions after randomisation, together with reasons	Figure 1
Recruitment	14a	Dates defining the periods of recruitment and follow-up	Section 2.1
	14b	Why the trial ended or was stopped	Section 2.1
Baseline data	15	A table showing baseline demographic and clinical characteristics for each group	Table 2
Numbers analysed	16	For each group, number of participants (denominator) included in each analysis and whether the analysis was by original assigned groups	Each table
Outcomes and estimation	17a	For each primary and secondary outcome, results for each group, and the estimated effect size and its precision (such as 95% confidence interval)	Each table / figure
	17b	For binary outcomes, presentation of both absolute and relative effect sizes is recommended	Not applicable
Ancillary analyses	18	Results of any other analyses performed, including subgroup analyses and adjusted analyses, distinguishing pre-specified from exploratory	Not applicable
Harms	19	All important harms or unintended effects in each group (for specific guidance see CONSORT for harms)	Section 2.5
		**Discussion**	
Limitations	20	Trial limitations, addressing sources of potential bias, imprecision, and, if relevant, multiplicity of analyses	Section 4, last paragraph

Generalisability	21	Generalisability (external validity, applicability) of the trial findings	Section 4, last paragraph
Interpretation	22	Interpretation consistent with results, balancing benefits and harms, and considering other relevant evidence	Section 4
**Other information**			
Registration	23	Registration number and name of trial registry	Section 2.16
Protocol	24	Where the full trial protocol can be accessed, if available	Not applicable
Funding	25	Sources of funding and other support (such as supply of drugs), role of funders	Funding statement

Table S2. Anthropometric gains through 4 months for the full analysis set [a].

	n	SYN (Mean ± SD)	CTRL (Mean ± SD)	BF (Mean ± SD)	p-value
Length gain (mm/week)	SYN = 94<br>CTRL = 97<br>BF = 65	7.9 ± 0.9	7.7 ± 1.0	7.3 ± 1.0	SYN vs BF p<0.001<br><br>CTRL vs BF p=0.017<br><br>SYN vs CTRL p=0.158
Head circumference gain (mm/week)	SYN = 95<br>CTRL = 97<br>BF = 65	4.1 ± 0.6	4.1 ± 0.7	3.8 ± 0.6	SYN vs BF p=0.001<br><br>CTRL vs BF p<0.001<br><br>SYN vs CTRL p=0.491

[a] BF, breastfed group; CTRL, control formula-fed group; SYN, experimental formula-fed group. Analyses were performed using propensity score adjusted ANCOVA, correcting for baseline value, sex, mode of delivery, and study center. All p-values were adjusted for multiple comparisons using Benjamini-Hochberg correction.

frontiers

Table S3. Stooling patterns through 4 months for the full analysis set [a].

Visit	SYN (Mean ± SD)	CTRL (Mean ± SD)	BF (Mean ± SD)	*p*-value		
				SYN vs BF	CTRL vs BF	SYN vs CTRL
Stool frequency per day						
≤ 14 days	2.8 ± 1.9	2.9 ± 1.9	4.7 ± 2.5	N.A.	N.A.	N.A.
1 mo	1.8 ± 1.1	1.9 ± 1.2	3.7 ± 2.6	0.006	0.143	0.143
2 mo	1.5 ± 1.2	1.4 ± 1.0	3.1 ± 2.5	0.016	0.028	0.774
3 mo	1.4 ± 1.2	1.1 ± 0.6	2.2 ± 1.6	0.020	0.009	0.509
4 mo	1.5 ± 1.3	1.3 ± 0.9	1.9 ± 1.3	0.002	0.001	0.662
Stool consistency						
≤ 14 days	0.9 ± 0.8	0.8 ± 0.7	0.4 ± 0.6	N.A.	N.A.	N.A.
1 mo	0.7 ± 0.6	0.8 ± 0.6	0.4 ± 0.4	0.037	0.037	0.812
2 mo	0.8 ± 0.6	0.7 ± 0.5	0.4 ± 0.5	0.285	0.579	0.360
3 mo	0.8 ± 0.5	0.7 ± 0.5	0.4 ± 0.6	0.044	0.216	0.216
4 mo	0.9 ± 0.6	0.8 ± 0.6	0.5 ± 0.6	0.181	0.405	0.413
	**SYN (Days, %)**	**CTRL (Days, %)**	**BF (Days, %)**	***p*-value**		
				**SYN vs BF**	**CTRL vs BF**	**SYN vs CTRL**
Difficulty in passing stools						
≤ 14 days	14 (13)	8 (8)	1 (1)	N.A.	N.A.	N.A.
1 mo	47 (24)	50 (24)	19 (14)	0.446	0.446	0.844
2 mo	46 (20)	37 (17)	20 (13)	0.798	0.798	0.798
3 mo	24 (10)	20 (9)	18 (11)	0.028	0.030	0.803
4 mo	14 (5)	19 (8)	11 (6)	0.501	0.493	0.501

[a] BF, breastfed group; CTRL, control formula-fed group; SYN, experimental formula-fed group; N.A., not available. Analyses were performed using propensity score adjusted ANCOVA, correcting for baseline value, age, and study center. All p-values were adjusted for multiple comparisons using Benjamini-Hochberg correction.

Table S4. Fecal short chain fatty acids (SCFAs) and lactic acid for the full analysis set at 3 months [a].

				p-value		
	**SYN (mean ± SD)**	**CTRL (mean ± SD)**	**BF (mean ± SD)**	**SYN vs. BF**	**CTRL vs. BF**	**SYN vs. CTRL**
Acetic acid (%)	72.9 ± 10.9	72.6 ± 12.1	85.4 ± 11.9	0.010	0.032	0. 327
Butyric acid (%)	8.7 ± 6.3	9.8 ± 5.0	5.1 ± 7.5	0.199	0.056	0.199
Propionic acid (%)	17.5 ± 9.1	16.5 ± 9.4	9.3 ± 8.7	0.005	0.078	0.078
Valeric acid (%)	0.9 ± 1.4	1.2 ± 2.7	0.2 ± 0.5	0.584	0.584	0.670
				p-value		
	**SYN (GM [GSD])**	**CTRL (GM [GSD])**	**BF (GM [GSD])**	**SYN vs. BF**	**CTRL vs. BF**	**SYN vs. CTRL**
DL-lactic acid (µmol/g)	0.8 (8.8)	0.7 (10.4)	7.0 (6.4)	0.002	0.002	0.849

[a] BF, breastfed group; CTRL, control formula-fed group; SYN, experimental formula-fed group; GM, geometric mean; GSD, geometric standard deviation. Percentages represent % of total SCFA; DL-lactic acid values were log-transformed. Values below the lower limit of quantification (LLOQ) were imputed by LLOQ/2 and values above the upper limit of quantification (ULOQ) were imputed by ULOQ. Analyses were performed using propensity score adjusted ANCOVA, correcting for baseline value, age, and study center. All p-values were adjusted for multiple comparisons using Benjamini-Hochberg correction.
